# Tracking inflammation resolution signatures in lungs after SARS-CoV-2 omicron BA.1 infection of K18-hACE2 mice

**DOI:** 10.1371/journal.pone.0302344

**Published:** 2024-11-12

**Authors:** Agnes Carolin, Kexin Yan, Cameron R. Bishop, Bing Tang, Wilson Nguyen, Daniel J. Rawle, Andreas Suhrbier

**Affiliations:** 1 QIMR Berghofer Medical Research Institute, Brisbane, Queensland, Australia; 2 GVN Centre of Excellence, Australian Infectious Disease Research Centre, Brisbane, Queensland, Australia; Lerner Research Institute - Cleveland Clinic, UNITED STATES OF AMERICA

## Abstract

The severe acute respiratory syndrome coronavirus 2 (SARS-CoV-2) causes Coronavirus Disease 2019 (COVID-19), which can result in severe disease, often characterised by a ‘cytokine storm’ and the associated acute respiratory distress syndrome. However, many infections with SARS-CoV-2 are mild or asymptomatic throughout the course of infection. Although blood biomarkers of severe disease are well studied, less well understood are the inflammatory signatures in lung tissues associated with mild disease or silent infections, wherein infection and inflammation are rapidly resolved leading to sequelae-free recovery. Herein we described RNA-Seq and histological analyses of lungs over time in an omicron BA.1/K18-hACE2 mouse infection model, which displays these latter features. Although robust infection was evident at 2 days post infection (dpi), viral RNA was largely cleared by 10 dpi. Acute inflammatory signatures showed a slightly different pattern of cytokine signatures compared with severe infection models, and where much diminished 30 dpi and absent by 66 dpi. Cellular deconvolution identified significantly increased abundance scores for a number of anti-inflammatory pro-resolution cell types at 5/10 dpi. These included type II innate lymphoid cells, T regulatory cells, and interstitial macrophages. Genes whose expression trended downwards over 2–66 dpi included biomarkers of severe disease and were associated with ‘cytokine storm’ pathways. Genes whose expression trended upward during this period were associated with recovery of ciliated cells, AT2 to AT1 transition, reticular fibroblasts and innate lymphoid cells, indicating a return to homeostasis. Very few differentially expressed host genes were identified at 66 dpi, suggesting near complete recovery. The parallels between mild or subclinical infections in humans and those observed in this BA.1/K18-hACE2 mouse model are discussed with reference to the concept of “protective inflammation”.

## Introduction

The severe acute respiratory syndrome coronavirus 2 (SARS-CoV-2) has caused a global pandemic of Coronavirus Disease 2019 (COVID-19) [1], with over 770 million cases and 7 million deaths [2,3]. Severe acute disease is primarily characterised by infection of the respiratory tract and pathological hyper-inflammatory responses, often referred to as a “cytokine storm” that can lead to acute respiratory distress syndrome (ARDS) and ultimately mortality [4–6]. Post-acute and chronic sequelae of COVID-19 are now well described, with post-COVID conditions affecting 10–30% of COVID-19 patients [7] and long-COVID affecting at least 10% of patients [8–10].

The inflammatory mediators that are associated with severe COVID-19, as distinct from mild disease, have been the focus of many studies, with such insights having the potential to identify therapeutic anti-disease interventions [4,6,11–18]. Elevated mRNA or protein levels of a range of cytokines have been identified in peripheral blood of severe COVID-19 patients, these include *inter alia* IL-6, IL-1, IL-2, IL-17, IL-18, TNF, IFNG and CSF2 (GM-CSF) (*ibid*). Elevated IL-10 in the peripheral blood has also been associated with severe disease, but may be useful later in the infection to suppress the hyper-cytokinemia associated with ARDS [19–21]. Elevated Th2 [11,22–24] and Th17 responses [11,25] have been associated with poor outcomes, with Th1/Th2 [26–28] and Th1/Th17 imbalances [29] also reported to be detrimental. T regulatory cells (Tregs) are viewed as providing protection against the cytokine storm [30], whereas neutrophils promote the latter and contribute to disease severity [11]. Monocytes and macrophages play key protective roles against SARS-CoV-2 infection, but depending on their differentiation and activation state, are also important contributors to over-production of inflammatory cytokines [31,32]. Higher viral loads appear to positively associate with COVID-19 severity [33,34], although this may be associated with more prolonged high viral loads in patients with severe disease [35].

An estimated 25–30% of SARS-CoV-2 infections are asymptomatic throughout the course of infection [36,37], with a constellation of factors potentially contributing [38–40]. Although fatal disease is associated with higher viral loads, asymptomatic individuals often do not have lower acute viral loads than symptomatic patients [41–44]. Humans have also been shown to tolerate a certain level of lower respiratory tract infection (and associated radiographic abnormalities), without reporting symptoms [45]. Persistent asymptomatics do, nevertheless, appear able to clear the virus more rapidly [46,47]. For instance, only minimal PCR-detectable viral RNA was present in saliva of a small cohort of such individuals by days 9–10 post first positive diagnosis (after daily saliva testing) [46]. Individuals with silent infections may have lower levels of circulating proinflammatory cytokines [47], although this is not a universal finding [48]. Silent or subclinical infections have been associated with powerful antiviral responses [49], early and robust innate immune responses [50], specific HLA genes [40], and specific gene expression patterns [51,52], such as higher plasma levels of the anti-inflammatory cytokines IL-10, IL-1RA and IL-19 [48]. Although in humans there can be long-term consequences of asymptomatic SARS-CoV-2 infections, these are significantly more prevalent after symptomatic infections [53].

Herein we provide the transcriptional profile of lung infection over time in a model of SARS-CoV-2 omicron BA.1 infection of K18-hACE2 mice, wherein the majority of mice showed no significant overt signs or symptoms of disease [54]. The acute phase was characterised by a significant lung infection and a pattern of proinflammatory cytokine signatures differing slightly from those reported previously [55]. By 10 days post infection (dpi), virus had been largely cleared and a series of cell types associated with damage control, suppression of inflammation, homeostasis and repair were identified by cellular deconvolution. Few differentially expressed genes (DEGs) were identified in lungs by 66 dpi, arguing that the model describes near complete resolution of the infection, without long-term pulmonary sequelae. The term “protective inflammation” has been coined to describe the inflammatory processes that lead to subclinical or mild disease, resulting in sequelae-free outcomes [56]. Protective responses are likely to have distinct gene expression patterns and modulated cellular infiltrates, when compared with inflammatory disease [40,57,58]. Herein we provide insights into the transcriptional signatures in lungs that characterise resolution of inflammation and return to homeostasis, and also identify a series of parallels with human studies.

## Materials and methods

### Ethics statements and regulatory compliance

Collection of nasal swabs from consented COVID-19 patients was approved by the QIMR Berghofer Medical Research Institute Human Research Ethics Committee (P3600). Patients self-nominated to provide samples and declared they had COVID-19, with no medical records accessed. Patients took their own nasal swab samples as per rapid antigen test. Patients signed a consent form that was countersigned by the study leader. Consent forms are held on file at QIMR Berghofer MRI. Samples were deidentified and virus isolated from the nasal swab material. All participants were adults with degree-level education. Signing of consent forms was not witnessed due to infection risk.

All mouse work was conducted in accordance with the Australian code for the care and use of animals for scientific purposes (National Health and Medical Research Council, Australia). Mouse work was approved by the QIMR Berghofer MRI Animal Ethics Committee (P3600). All infectious SARS-CoV-2 work was conducted in the BioSafety Level 3 (PC3) facility at the QIMR Berghofer MRI (Department of Agriculture, Fisheries and Forestry, certification Q2326 and Office of the Gene Technology Regulator certification 3445). Breeding and use of GM mice was approved under a Notifiable Low Risk Dealing (NLRD) Identifier: NLRD_Suhrbier_Oct2020: NLRD 1.1(a). Mice were euthanized using carbon dioxide.

### The SARS-CoV-2 isolates

The BA.1 omicron isolate was obtained at QIMR Berghofer MRI from nasal swabs from a consented COVID-19 patient by culture in Vero E6 cells (ATCC C1008). The omicron BA.1 isolate, SARS-CoV-2_QIMR01_ (SARS-CoV-2/human/AUS/QIMR01/2022), belongs to the BA.1.17 lineage (GenBank: ON819429 and GISAID EPI_ISL_13414183) [59,60]. BA.1 viral stocks were propagated in Vero E6 cells as described [61]. UV inactivation of the BA.1 virus was undertaken using the UVC 500 Ultraviolet Crosslinker (Hoefer) (dose 7650 J/m^2^ UVC) as described [61]. Virus stocks were titered using cell culture infectious dose 50% (CCID_50_) assays [62].

The original strain isolate, SARS-CoV-2_QLD02_, (hCoV-19/Australia/QLD02/2020) (GISAID accession EPI_ISL_407896) was kindly provided by Dr. Alyssa Pyke (Queensland Health Forensic &Scientific Services, Queensland Department of Health, Brisbane, Australia). Propagation and titration was undertaken as for BA.1.

### K18-hACE2 mice, infection and monitoring

Heterozygous K18-hACE2 mice (strain B6.Cg-Tg(K18-ACE2) 2Prlmn/J, JAX Stock No: 034860) were purchased from The Jackson Laboratory, USA, and were maintained in-house as heterozygotes by backcrossing to C57BL/6J mice (Animal Resources Center, Canning Vale WA, Australia). Heterozygotes were inter-crossed to generate homozygous K18-hACE2 mice (on a C57BL/6J background [63]) as described [54]. Mice were held under standard animal house conditions (for details see [59]) and homozygous female mice received intrapulmonary infections delivered via the intranasal route with 5×10^4^ CCID_50_ of virus in 50 μl RPMI 1640 whilst under light anesthesia. Mice were anesthetized with 4% isoflurane (Piramal Enterprises Ltd., Andhra Pradesh, India) administered using The Stinger, Rodent Anesthesia System (Advanced Anaesthesia Specialists/Darvall, Gladesville, NSW, Australia), oxygen flow rate 0.8 L/min. Mice were placed individually into the induction chamber and after loss of righting reflex (≈30 sec) the mice were kept in the chamber for 2 mins before intranasal administration of virus. For each group for each time point, a comparable mean age and age distribution of mice was used (mean age 26. 3 + SD 1.1. weeks). Mice were weighed and monitored as described [54,64]. Mice were euthanized using CO_2_, lungs were removed, with the left lung fixed in formalin for histology, the right lung inferior lobe placed in RNAlater for RNA-Seq and RTqPCR, and the remaining lobes used for tissue titers determination by CCID_50_ assays using Vero E6 cells as described [62,64].

### CCID_50_ assays

Tissue titers were determined as described [61]. Briefly, 5 fold serial dilutions of clarified tissue homogenates were applied in duplicates to Vero E6 cells in 96 well plates. After 6 days cytopathic effects were observed by inverted light microscope [62]. The virus titer was determined by the method of Spearman and Karber [65].

### SARS-CoV-2 genome copy number determination

The right inferior lung lobes were harvested and placed in RNAlater (Invitrogen). Samples were transferred to TRIzol (Life Technologies) and were homogenized twice at 6000 x g for 15 sec (Precellys 24 Homogenizer, Bertin Instruments, Montigny-le-Bretonneux, France) as described [64]. Homogenates were centrifuged at 14,000 × g for 10 min and RNA was isolated as per manufacturers’ instructions. cDNA was synthesized using ProtoScript II First Strand cDNA Synthesis Kit (New England Biolabs) and qPCR performed using iTaq Universal Probes Kit (Bio-Rad). Lung samples were obtained at 2 and 5 dpi from K18-hACE2 mice infected with BA.1 or an original strain isolate (SARS-CoV-2_QLD02_). Primers that span the junction between Orf1a and Orf1b were used in order to measure genomic, rather than subgenomic RNA levels [66]; F 5ʹ-GGCCAATTCTGCTGTCAAATTA-3ʹ, R 5ʹ-CAGTGCAAGCAGTTTGTGTAG-3ʹ. Primers for the house keeping gene, mRPL13a, were 5ʹ-GAGGTCGGGTGGAAGTACCA-3ʹ and 5ʹ-TGCATCTTGGCCTTTTCCTT-3ʹ [67]. PCR fragments of SARS-CoV-2 Orf1ab and mRPL13a were gel purified and 10-fold serial dilutions of estimated copy numbers were used as standards in qPCR to calculate copies in samples reactions. SARS-CoV-2 Orf1ab copies were normalized by mRPL13a copy number in each reaction. qPCR reactions were performed in duplicate and averaged to determine the copy number in each sample.

### Histology

Lungs were fixed in 10% formalin, embedded in paraffin, and sections stained with H&E (Sigma Aldrich). Slides were scanned using Aperio AT Turbo (Aperio, Vista, CA, USA). Areas with overt leukocyte infiltrates (with high focal densities of dark purple staining nuclei) were measured manually using the “Pen Tool” in the Aperio ImageScope software v10 (Leica Biosystems, Mt Waverley, Australia). White space analysis was undertaken using QuPath v0.2.3 [68].

### Immunohistochemistry

Immunohistochemistry was undertaken as described using the anti-SARS-CoV-2 spike monoclonal antibody, SCV2-1E8 [60], except that the monoclonal (IgG2a) was purified using Protein A affinity chromatography and applied to sections at 4 μg/ml for 1 hr.

### RNA isolation, library preparation, RNA-Seq and bioinformatics

RNA isolation, library preparation and RNA-Seq was undertaken as described [55,69]. Briefly, lung tissues were harvested into RNAlater, RNA was extracted using TRIzol (Life Technologies), and RNA concentration and quality measured using TapeStation D1kTapeScreen assay (Agilent). cDNA libraries were generated using Illumina TruSeq Stranded mRNA library prep kit. RNA-Seq was undertaken as described using Illumina Nextseq 2000 platform generating 75 bp paired end reads [55]. Mean quality scores were above Q20 for all samples. Mouse RNA-Seq reads were aligned to a combined mouse (GRCm39, version M27) and SARS-CoV-2 BA.5 reference genome [54] using STAR aligner. Viral read counts were generated using Samtools v1.16. Read counts for host genes were generated using RSEM v1.3.1. Genes with low read counts were identified and removed separately for each time point using DESeq2 v1.40.2. Differentially expressed host genes were identified using DESeq2 using a FDR cut-off of q<0.05. Pathway analysis was performed with host DEGs using Ingenuity Pathway Analysis (IPA, v84978992) (QIAGEN) using the Canonical pathways, Up-Stream Regulators (USR) and Diseases or Functions features as described [63]. Gene Set Enrichment Analyses (GSEAs) were undertaken using GSEA v4.1.0 with gene sets provided in MSigDB (≈ 45,000 gene sets) and in the Blood Transcription Modules [70], and gene lists generated using DESeq2 ranked by log_2_ fold-change. Relative abundances of cell types were estimated in R v4.1.0 from RSEM ‘expected counts’ using SpatialDecon v1.4.3 [71] and cell-type expression matrices obtained from the “Mouse Cell Atlas Lung Cell expression matrix” and the “NanoString Immune Cell Family expression matrix”.

### RTqPCR validation of RNA-Seq data

The RTqPCR was conducted as described [69]. Briefly, cDNA was synthesized from total RNA with ProtoScript II First Strand cDNA Synthesis Kit (New England Biolabs) and qPCR performed using iTaq Universal SYBR Green Supermix (Bio-Rad) as per manufacturer’s instructions with primers (Integrated DNA Technologies) for mouse *Oas3* (Forward 5’-TGGCAATCCCATCAAGCCAT-3’ and Reverse 5’- CTGAGGGCTGGTGTCACTTT-3’), *Irf7* (Forward 5’-ACCGTGTTTACGAGGAACCC-3’ and Reverse 5’-GTTCTTACTGCTGGGGCCAT-3’), *Ccl8* (Forward 5’- GGGTGCTGAAAAGCTACGAGAG -3’ and Reverse 5’- GGATCTCCATGTACTCACTGACC -3’). qPCR reactions were performed in duplicate and averaged. Gene expression was normalized with the house-keeping gene, *mRpl13a* [67]. The 2-ΔΔCt method was used to calculate the log_2_ fold-change [72].

### Statistics

The t-test (with Welch’s correction) was used if the difference in variances was <4 fold, skewness was > - 2 and kurtosis was <2. The t test significance and variance were determined using Microsoft Excel. Skewness and kurtosis were determined using IBM SPSS Statistics for Windows v19.0 (IBM Corp., Armonk, NY, USA). Otherwise, the non-parametric Kolmogorov-Smirnov exact test was performed using GraphPad Prism 10.

## Results

### SARS-CoV-2 omicron BA.1 provided robust lung infection in K18-hACE2 mice that was cleared by 10 dpi

**Fig 1:**
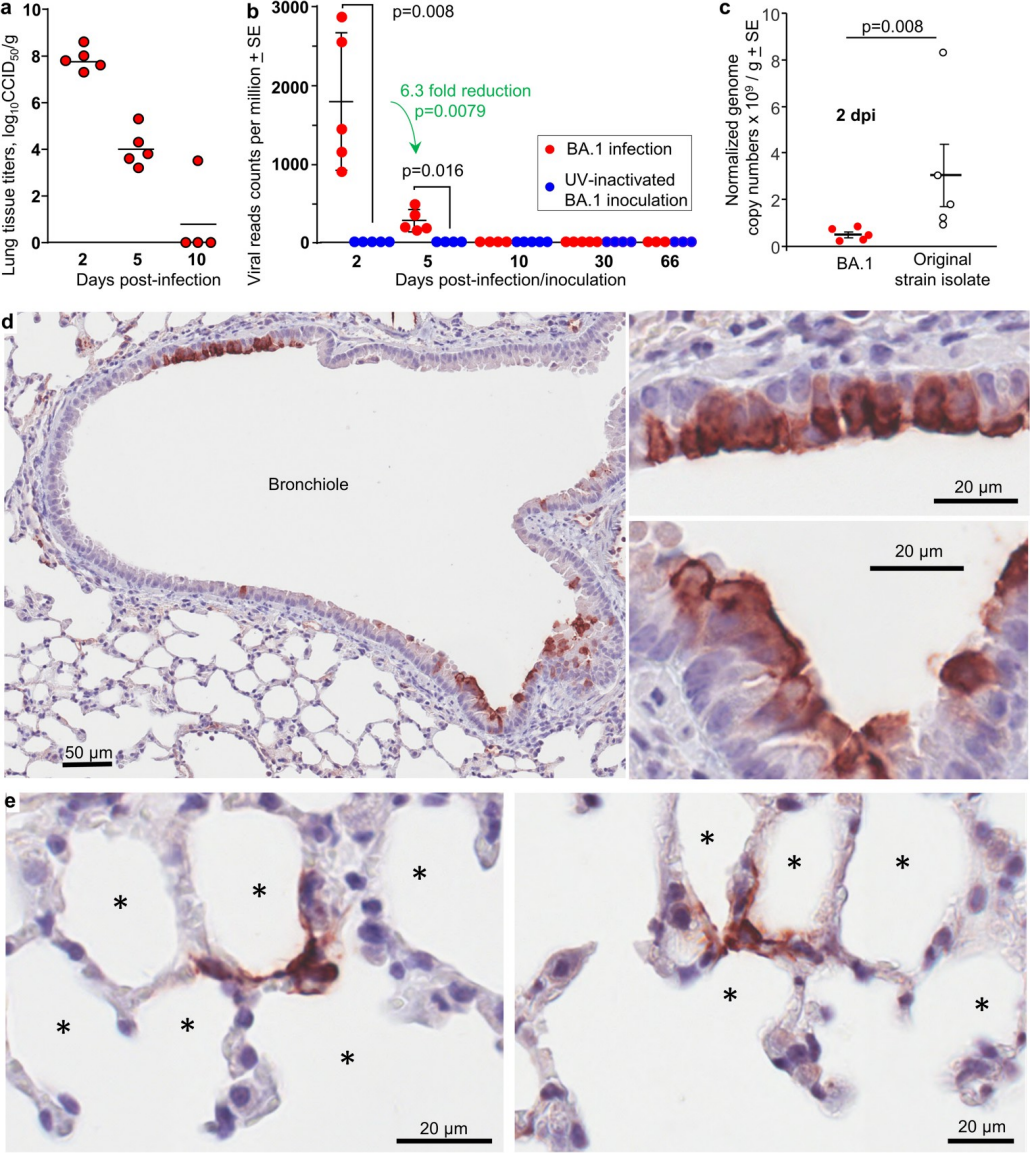
Viral loads and immunohistochemistry of viral antigen in lungs of BA.1 infected K18-hACE2 mice. **a** Lung tissue titers at the indicated days post infection with BA.1 (limit of detection per mouse ≈ 2 log_10_CCID_50_/g). **b** Viral read counts per million reads, determined by RNA-Seq for K18-hACE2 mice infected with BA.1 or inoculated with UV-inactivated BA.1 virus. Statistics by Kolmogorov Smirnov exact tests. **c** Viral genome copy number determined by RTqPCR for K18-hACE2 mice infected with BA.1 or an original strain isolated SARS-CoV-2_QLD02_. Statistics by Kolmogorov-Smirnov exact test. **d** Immunohistochemistry of BA.1-infected lungs at 2 dpi, stained with an anti-spike monoclonal antibody. Staining of bronchial epithelia cells (dark brown) can be seen top left and bottom right (left image), with enlargements of these two areas shown in the right 2 images. **e** As for d showing staining of cells that surround alveoli with morphology consistent with that of pneumocytes (alveolar epithelial cells). * alveolar air sacs.

K18-hACE2 mice were given an intrapulmonary inoculum via the intranasal route of SARS-CoV-2 BA.1 (5x10^4^ CCID_50_) or the same inoculum of UV-inactivated BA.1 virus. Lungs were harvested at 2, 5, 10, 30 and 66 days post infection (dpi) and tissue titers (Fig 1A), RNA-Seq (Fig 1B and see below) and histology (see below) undertaken. Lung viral titers reached a mean titer of 7.86 + SD 0.49 log_10_CCID_50_/g on 2 days post infection (dpi), falling to a mean of 4.04 + SD 0.8 log_10_CCID_50_/g at 5 dpi, and by 10 dpi, 3 of 4 BA.1 infected mice showed no detectable virus titers (Fig 1A). Viral read counts from lungs of BA.1 infected mice, obtained from RNA-Seq data, showed a similar trend, with no significant viral reads detected at 30 and 66 dpi (Fig 1B). No significant read counts were obtained from mice inoculated with UV-inactivated virus (Fig 1B). Although different lung lobes were used for viral titrations and for RNA-Seq, there was a highly significant correlation between these two measures of viral load (S1A Fig in S1 File).

When compared with infection with an original strain isolate (and using the same assay systems) [55,64], infectious viral titers for BA.1 were comparable at 2 dpi, but were ≈ 2 logs lower at 5 dpi (Fig 1A). Again using the same assay systems, mean viral reads counts (Fig 1B), representing genomic and subgenomic viral RNA, were ≈ 2 logs higher in original strain infected K18-hACE lungs at 2 dpi [55], and did not diminish by more than 0.75 logs by 4 and 7 dpi [55,73]. Mean lung viral genome copy numbers [66] in K18-hACE2 mice were also ≈ 6 fold (0.8 log) lower after BA.1 infection than after infection with an original strain isolate (SARS-CoV-2_QLD02_) (Fig 1C). Viral replication and/or transcription was thus lower in BA.1 vs. original strain infected mice, and diminished more rapidly.

As reported previously [54,74,75], most mice in the BA.1/K18-hACE2 model do not develop a brain infection. Brain infection is associated with >20% weight loss and an ensuing ethical requirement for euthanasia (S1B Fig in S1 File). After infection of K18-hACE2 mice with original strain isolates, mice succumb to brain infections between 4 and 7 dpi [54,64]. This BA.1 model thus provides a significant lung infection, and permits analysis of long-term responses to SARS-CoV-2.

### BA.1 infected bronchial epithelial cells and pneumocytes in K18-hACE2 mouse lungs

Immunohistochemistry (IHC) of lungs taken 2 dpi (peak viral load), using an anti-spike monoclonal antibody [60], illustrated clear staining of bronchial epithelial cells (Fig 1D) and cells surrounding alveoli that have a morphology consistent with pneumocytes (Fig 1E). The BA.1/K18-hACE2 model thus recapitulates the main cell tropisms seen in human lung tissues, with infection of bronchial and alveolar epithelial cells by omicron variants well described [76,77].

### BA.1 induced histopathological lesions in lungs resolved by 66 dpi

**Fig 2:**
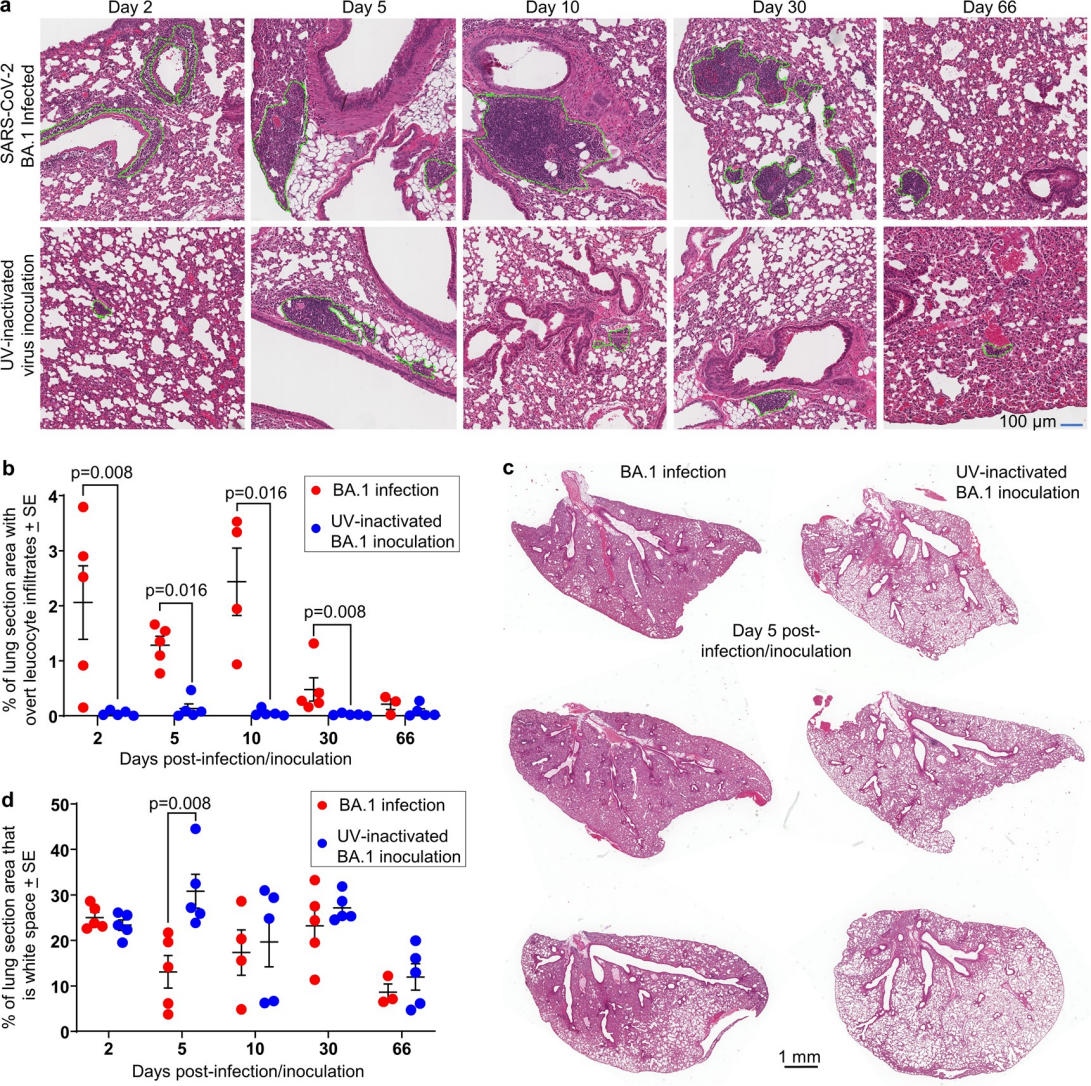
Histology of lungs after BA.1 infection of K18-hACE2 mice. **a** H&E staining of lung tissue sections at the indicated days after infection/inoculation. Green dotted lines encircle overt foci of cellular infiltrates. **b** Quantitation of cellular infiltrates. All cellular infiltrates were marked as in “a” and the summed infiltrate areas expressed as a percentage of the lung section area for each mouse. Statistics by Kolmogorov Smirnov exact tests. (For BA.1 infected mice, data for 2, 5 and 10 dpi are not significantly different, while 30 dpi shows a significant reduction from 5 and 10 dpi by t tests, p = 0.0.019 and p = 0.039, respectively). **c** Whole H&E stained lung sections from day 5 after infection/inoculation, illustrating reduced white space in lungs from infected mice. **d** QuPath digital analysis of white space in H&E stained whole lung sections such as those shown in “c”. Statistics by t test.

Hematoxylin and eosin (H&E) stained sections of lung tissues from BA.1 infected K18-hACE2 mice illustrated a series of histopathological features (Figs 2 and S2 in S1 File) previously identified in SARS-CoV-2 mouse models [78], although they appeared less severe than those seen after infection of K18-hACE2 mice with original strain isolates, consistent with previous reports [74,75]. For instance, in the latter, pronounced sloughing of the bronchial epithelium and fulminant occlusions of bronchi (with serum and red blood cells) were observed [79,80].

In BA.1 infected mice (as in other models [59,78]), focal areas of leucocyte infiltration were clearly evident, characterized by dense clusters of dark-purple staining leukocyte nuclei; although the infiltrates were more diffuse at 2 dpi (Fig 2A, green dashed lines). These areas were measured manually using the Aperio ImageScope “Pen Tool” (Fig 2A, green dashed lines), providing quantitation of leukocyte infiltration. Significantly higher levels of infiltration were seen for BA.1-infected versus UV-inactivated BA.1-inoculated mice at all time-points except 66 dpi, with infiltrates also substantially reduced by 30 dpi (Fig 2B) (Aperio Positive Pixel Count assessment of leukocyte infiltrates also showed significantly higher infiltrate levels at 5 dpi; S1c Fig in S1 File). Lung consolidation with resulting loss of white space, primarily as a result of diminished alveolar air sack volumes, was also clearly seen at 5 dpi (Fig 2C). Significant loss of white space at 5 dpi was confirmed by image analysis of scanned lung sections using QuPath (Fig 2D). Minor intrusion of red blood cells and serum into bronchi, and some alveolar edema were also occasionally observed in BA.1-infected mice (S2 Fig in S1 File). H&E images from PBS and naïve control lungs are shown in S3 Fig in S1 File.

In summary, lungs of BA.1 infected K18-hACE2 mice displayed some typical acute histopathological features described previously for SARS-CoV-2 infections, although less severe than after infection with original strain isolates. At 30 dpi BA.1 infection-associated histopathology was clearly resolving, and by 66 dpi had largely resolved.

### Cell types associated with resolution of inflammation were evident on 5 and 10 dpi

BA.1 infected lungs and UV BA.1 inoculated lungs were compared at each time point (2, 5, 10, 30 and 66 dpi) by RNA-Seq, using DESeq2 [81]. Full gene lists and bioinformatic analyses are provided for 2 dpi (S1 Table), 5 dpi (S2 Table), 10 dpi (S3 Table), 30 dpi (S4 Table), and 66 dpi (S5 Table). RTqPCR was undertaken for 3 genes and two time points to validate the RNA-Seq data, with highly significant concordance emerging for the two methods (S4a Fig in S1 File). We have previously reported high levels of concordance between these two methods in other studies [61,69].

**Fig 3:**
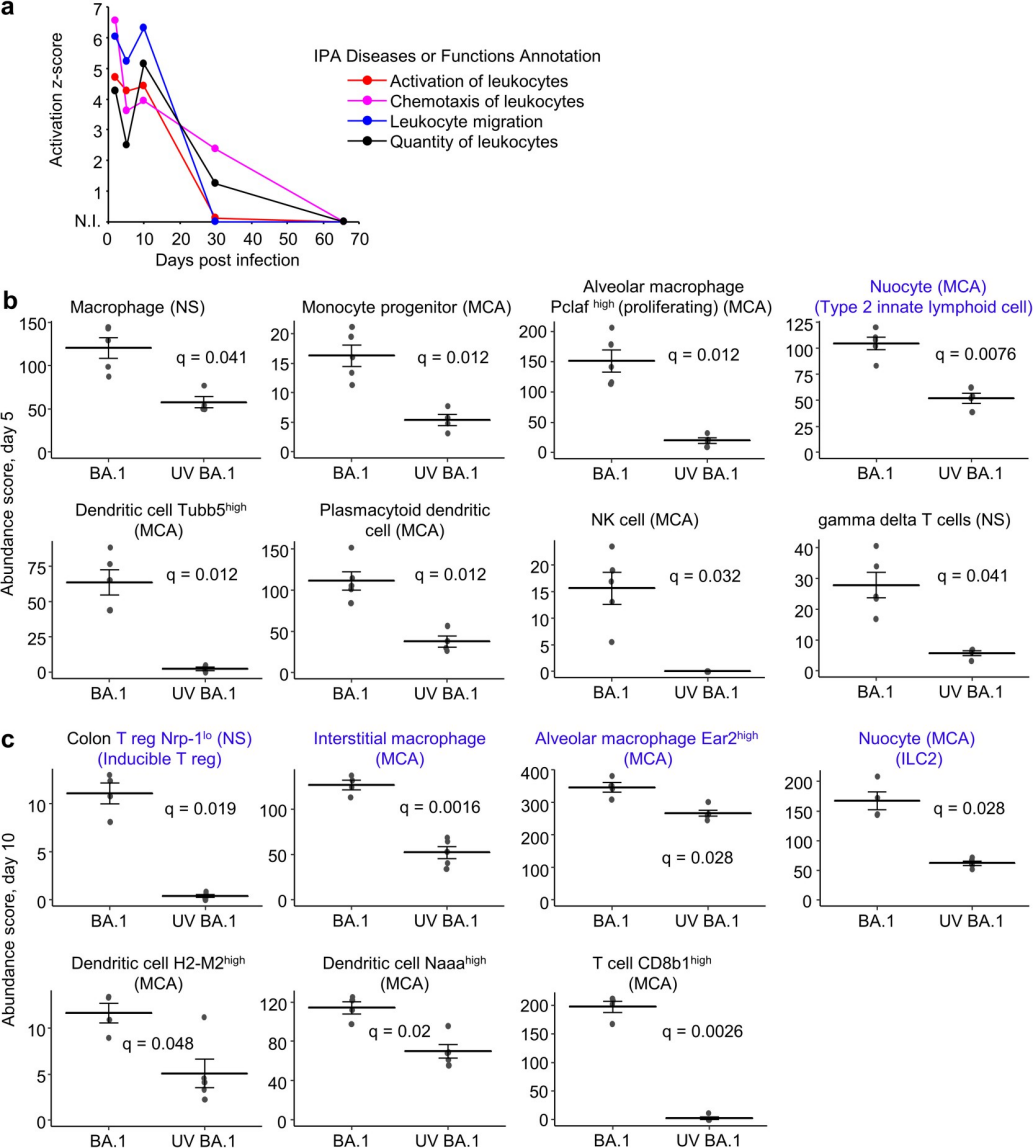
Cellular infiltrates and types identified by RNA-Seq of lungs for 2–30 dpi. **a** Z scores for the indicated annotations from Ingenuity Pathway analysis (IPA) Diseases or Function analysis plotted against dpi. (N.I. not identified; for 66 dpi there were insufficient DEGs for IPA analysis). **b** Using cellular deconvolution (SpatialDecon) and the gene expression matrices from the “Mouse Cell Atlas (MCA) Lung Cell expression matrix” and the “NanoString (NS) Immune Cell Family expression matrix”, cell types whose abundance scores were significantly different on day 5 were identified (q values provided). Blue text indicates cell type associated with inflammation resolution. **c** As for b but for day 10. Blue text indicates cell type associated with inflammation resolution. We assume “colon” T reg are well annotated in the NS matrices (and are thus readily identified), and represent tissue T regs, rather than implying that colonic T regs have migrated to the lungs.

Ingenuity Pathway analysis (IPA) Diseases or Functions annotations for Activation of leukocytes, Chemotaxis of leukocytes, Leukocyte migration and Quantity of leukocytes showed a progressive decline over time, with a slight elevation 10 dpi (Fig 3A), consistent with Fig 2B. To gain insights into the type of cellular infiltrates present in the lungs over time post infection with BA.1, cellular deconvolution (SpacialDecon) analysis using full gene sets (S1–S5 Tables) were used together with the gene expression matrices from the “Mouse Cell Atlas (MCA) Lung Cell expression matrix” and the “NanoString (NS) Immune Cell Family expression matrix”. As might be expected [82,83], a series of innate mononuclear leukocyte populations were identified at 5 dpi, which included macrophages, monocytes, dendritic cells, and NK cells, as well as gamma delta T cells [84] (Fig 3B). Increased abundance of nuocytes, ILC2 cells, was also identified on both 5 and 10 dpi (Fig 3B and 3C, nuocytes). These innate effector leukocytes mediate type-2 immunity [85] and are involved in the initiation of inflammation, but also have key roles in damage control, homeostasis and repair [86,87]. Lower frequencies of ILC2 cells have been associated with severe COVID-19 [88]. An increase abundance score for pericytes was also identified in infected lungs at 5 dpi (but not other times) (S4b Fig in S1 File). Pericytes express ACE2 and binding of spike protein has been shown *in vitro* to induce profound transcriptional changes in these cells [89,90], which may explain this result.

At 10 dpi, in addition to nuocytes, significantly increased abundance of three more cell types associated with repair and return to homeostasis was evident (Fig 3C, blue text). Inducible T regulatory cells (T regs) characterised by low neurophilin expression (Nrp-1^lo^) [91] were more abundant in infected lungs. T regs secrete *inter alia* IL-10 and are generally protective [92], not just by dampening proinflammatory responses, but also via prevention of fibrosis and maintenance of tissue homeostasis [93]. Interstitial macrophages were also elevated in infected lungs, with these cells having an inherently anti-inflammatory phenotype, secreting *inter alia* IL-10 (USR z score 1.69, q = 1.39E-29, S3 Table) and supporting T regs in the lung [94]. An Ear2+ alveolar macrophage population was also identified, with Nra4a1+, Ear2+ macrophages recently found to have a novel reparative phenotype [95]. Although perhaps proinflammatory, CD8 T cells were also more abundant at 10 dpi (Fig 3C), with CD8 T cells recently identified as key to protection against SARS-CoV-2 [96].

### Cytokine signatures in BA.1 infected lungs and their resolution by 30–66 dpi

**Fig 4:**
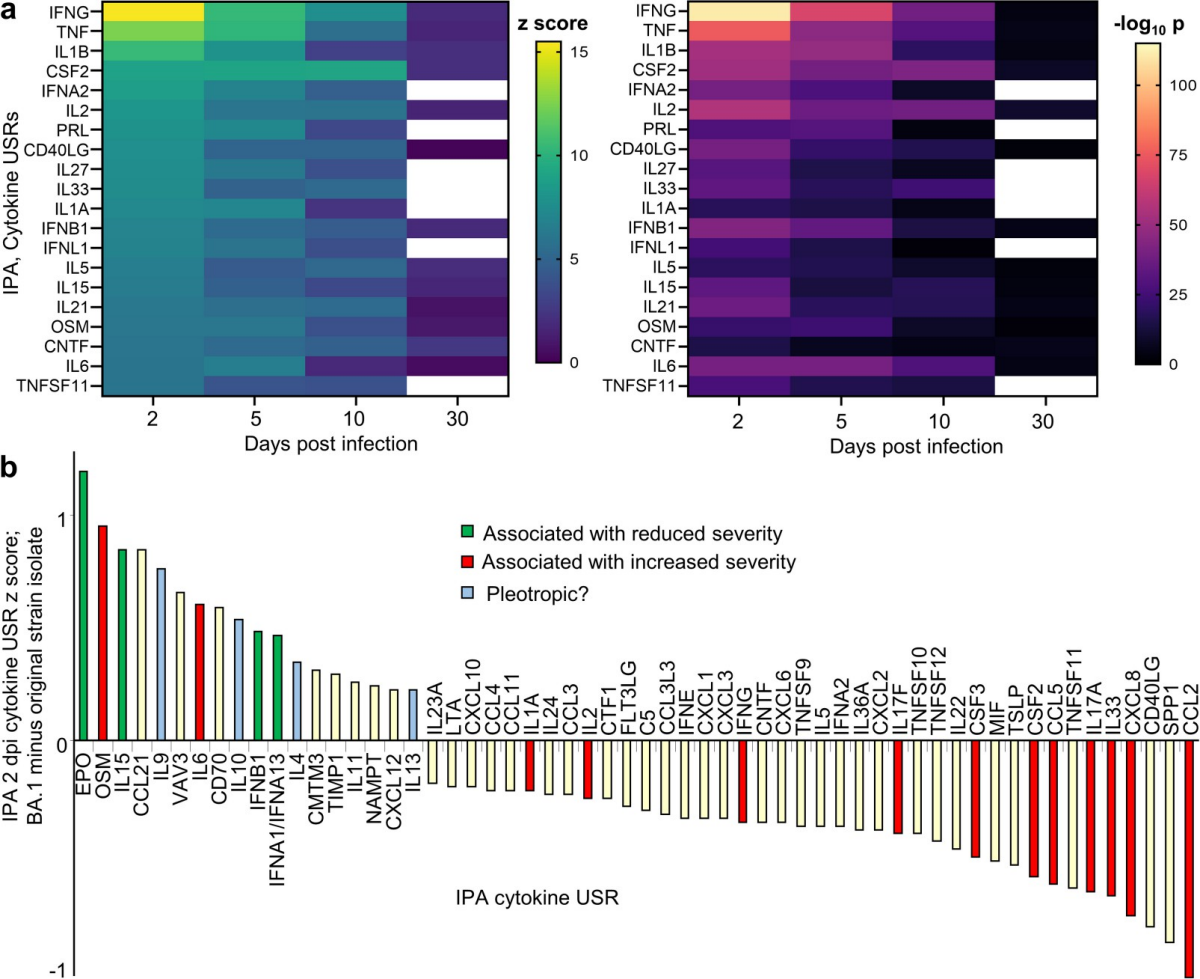
Inflammatory pathways identified by RNA-Seq of lungs for 2–30 dpi. **a** Heat maps of cytokine UpStream Regulator (USR) z scores and significance (p values, all p<0.05) determined by IPA. White boxes means no cytokine USR annotation was returned for 30 dpi by IPA. RNA-Seq identified too few DEGs on 66 dpi for IPA analysis. **b** IPA cytokine USRs from BA.1 infected K18-hACE2 mice (2 dpi) were compared with those previously reported for the more severe infection of K18-hACE2 mice by an original strain isolate [55]. Differences in cytokine z scores for 2 dpi (with a cutoff filter of >0.2 and < -0.2) are plotted. BA.1 infected lungs show slightly increased z scores for some cytokine signatures associated with reduced disease severity (green) and slightly reduced z scores for some cytokine signatures associated with increased disease severity (red).

Differentially expressed genes (DEGs, q<0.05) (S1–S5 Tables) were analyzed by IPA, with cytokine up-stream regulator (USR) z scores and p values illustrated by heat maps (Fig 4A). The acute cytokine responses were broadly comparable to those published previously for a K18-hACE2 mouse model of SARS-CoV-2 infection with an original strain isolate; specifically, hCoV-19/Australia/QLD02/2020 with the same viral dose of 5x10^4^ CCID_50_, the same route and volume of inoculation, and also female mice (NCBI SRA Bioproject PRJNA767499) [55]. However, when cytokine USR z scores at 2 dpi from BA.1 infection (Fig 4A) were compared with those from the aforementioned infection with the original strain isolate [55], small differences in z scores (between ≈1 and -1) emerged (Fig 4B). A series of USRs associated with less severe disease had slightly higher z score in BA.1 infected mice; these included EPO (erythropoietin) [97], IL-15 [98], and increased type I interferon responses [18,50] (Fig 4B). The z score was also higher for IL-10, with IL-10 elevated in severe COVID-19, but also able to suppress hyper-cytokinemia [19–21]. Elevated IL-4, IL-9 and IL-13 have been associated with more severe disease [22,23,99], although IL-9, IL-13 (and IL-5) are produced by type 2 innate lymphoid cells (ILC2 cells) [100] (see below), and increased IL-4 and IL-13 signatures may (in this context) reflect a relatively more balanced Th1/Th2 response [26,27]. Elevated OSM and IL-6 are also associated with more severe disease [18,101]. Another group of cytokines/chemokines associated with increased disease severity (IL-2, IFNG, IL-17, CSF2, CCL5, CXCL8, CCL2 [11,102,103] showed slightly lower z scores for BA.1 infected lungs (Fig 4B). IL-33 may drive fibrosis [104] and also showed a lower USR z score (Fig 4B).

Importantly, the cytokine response signatures had substantially abated by 30 dpi (Fig 4A), with the small number of DEGs on day 66 insufficient for meaningful pathway analysis. In addition, few, if any, upregulated inflammatory DEGs were identified at 66 dpi (see below).

### Transcriptional responses 66 dpi reflected adaptive immunity and resolution

**Fig 5:**
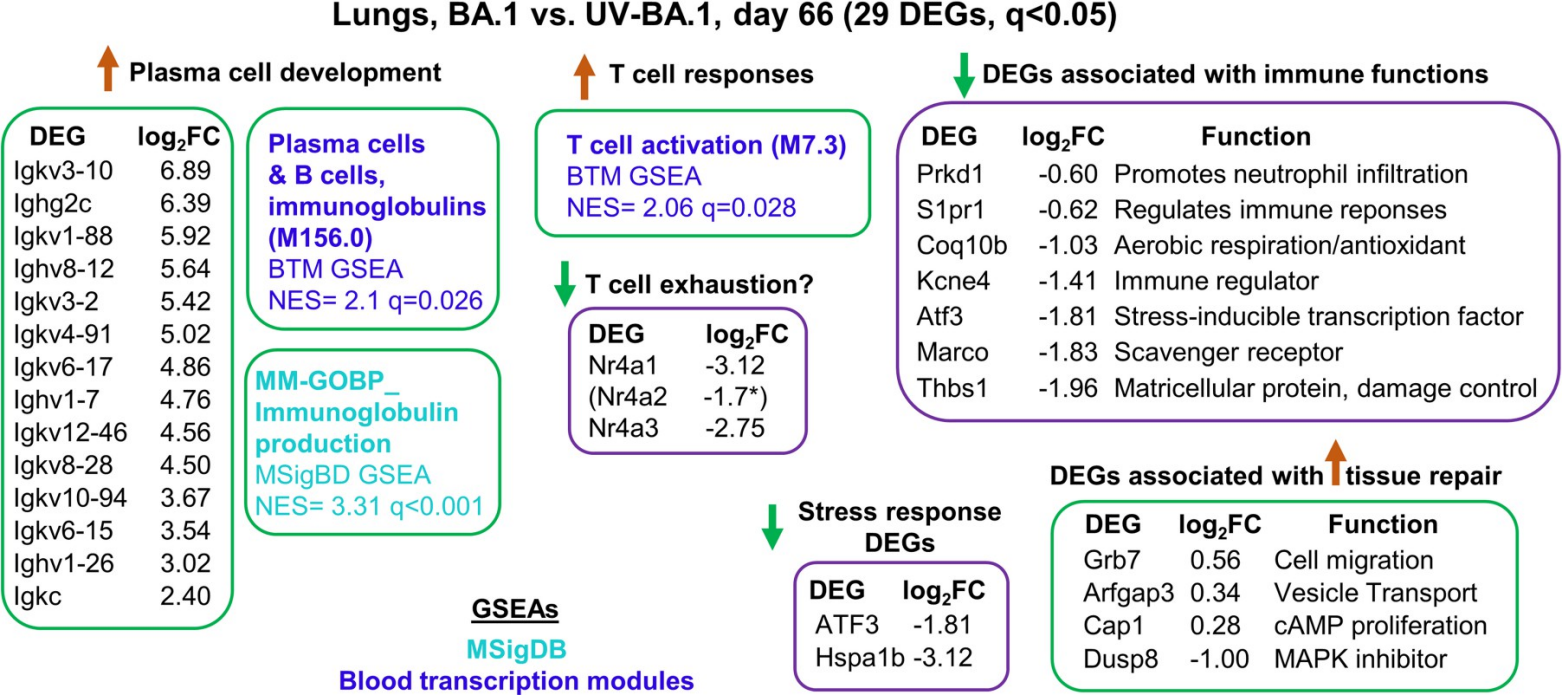
DEGs identified for 66 dpi. RNA-Seq data for day 66 dpi, with DEGs clustered by broad functions. The whole gene list was also interrogated by GSEAs using gene sets from MSigDB and Blood transcription modules.

The RNA-Seq data sets and bioinformatic analyses comparing lungs from BA.1 infected mice 66 dpi with mice 66 days after inoculation with UV-inactivated virus (S5 Table) are summarized in Fig 5. Only 29 DEGs were identified, of which 14 were immunoglobulin genes. This represents an insufficient number of DEGs for meaningful pathway analyses. DEGs were thus clustered into several groupings based on function (Fig 5, described below). A plot of the first two principle components of normalized Variance Stabilizing Transformation (VST) counts from all experimental groups indicated that the relatively small number of DEGs for day 66 was due to limited separation between BA.5 infected and UV control groups (S5 Fig in S1 File).

Most of the upregulated DEGs in lungs at 66 dpi were immunoglobulin genes (Fig 5) consistent with a more rigorous development of an adaptive immune response in BA.1 infected mice compared with mice inoculated with UV-inactivated virus. GSEAs also illustrate the development of T cell responses, with down-regulation of Nr4a family members potentially suggesting avoidance of T cell exhaustion [105]. However, the three Nr4a genes have a number of roles in T cell biology [106], and also have a diverse set of other activities in *inter alia* macrophages [95,107] and epithelial cells [69].

The three upregulated, non-Ig genes, can be associated with wound healing/tissue repair; Grb7 (Growth factor receptor-bound protein 7) [108], Arfgap3 (ADP-ribosylation factor GTPase-activating protein 3) [109,110], and Cap1 (cyclase-associated protein 1) [111]. In addition, Dusp8 (Dual specificity phosphatase 8) was down-regulated, with Dusp8 protein involved in negative regulation of MAP kinase superfamily members; thus Dusp down-regulation would promote cellular proliferation and differentiation [112].

Many of the remaining down-regulated genes have roles in various immune activities (Fig 5): Prkd1 (Serine/threonine-protein kinase D1), involved in *inter alia* promoting neutrophil infiltration [113]; S1pr1 (Sphingosine-1-phosphate receptor 1), essential for immune cell trafficking [114]; Coq10b, required for coenzyme Q function [115,116]; Kcne4 (Potassium voltage-gated channel subfamily E member 4), an immune regulator via modulation of Kv1.3 [117]; Atf3, a stress-inducible transcription factor [118]; Marco, a scavenger receptor [119]; and Thbs1 (thrombospondin), a matricellular protein involved in lung inflammation resolution and repair [120]. Two stress-inducible genes, Atf3 and Hspa1b (a heat shock protein 70 member) were down-regulated, with Atf3 and Hsp70 upregulated in a large range of stress responses [69,118].

In summary, at 66 dpi there were very few DEGs, with no indication of ongoing inflammation or tissue dysregulation, with identification of only a small number of upregulated genes that could be associated with wound healing processes.

### Genes whose expression positively correlated with dpi were associated with ciliated epithelial cells and protein synthesis

Using the complete gene sets (S1–S5 Tables), genes were identified whose expression showed significant positive correlation with dpi (Pearson correlations, FDR corrected p, q<0.05). Genes whose expression also showed significant positive correlation with days post inoculation of UV-inactivated virus were excluded from the analysis, thereby retaining only genes associated with active infection. Immunoglobulin genes were also removed as the development of immune responses is understood and established. The process identified 2786 genes (S6 Table).

**Fig 6:**
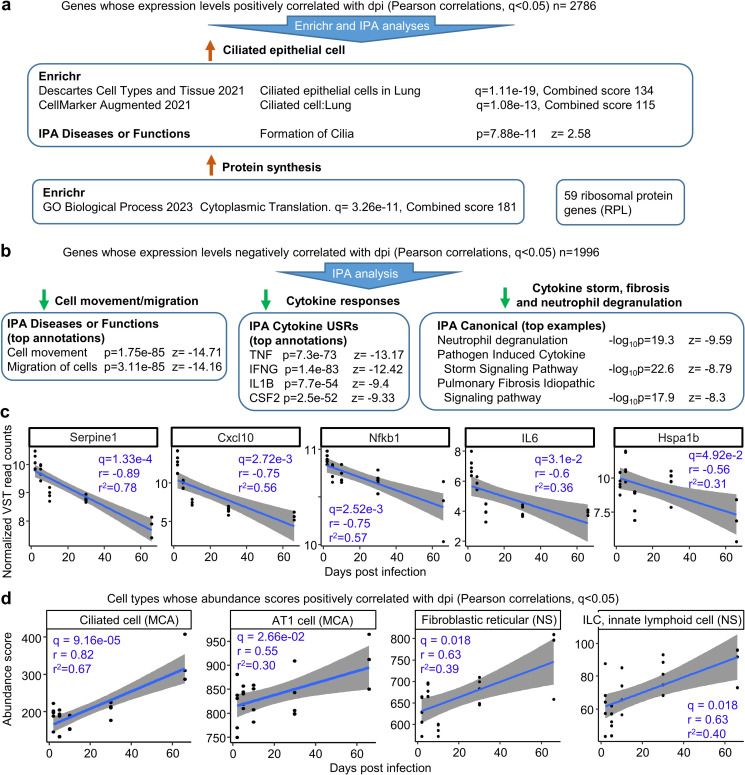
Genes whose expression correlated with dpi. **a** Using the whole gene lists, genes whose expression showed a statically significant positive correlation with dpi were identified. After removing genes that also positively correlated with dpi in the UV-inactivated BA.1 inoculated control groups, 2786 genes remained. The 2786 genes were analyzed by Enrichr and IPA (for the latter the r value was entered in the log2 expression column). Significant results clustered into two categories ‘Ciliated epithelial cell’ and ‘Protein synthesis’. Of the 2786 genes, 59 were RPL (ribosomal protein) genes. **b** Using the whole gene lists, genes whose expression showed a statically significant negative correlation with dpi were identified. After removing genes that also negatively correlated with dpi in the UV control groups, 1996 genes remained. The 1996 genes were analyzed by IPA as above, with some top annotations shown. **c** Normalized expression data (VST ‐ Variance Stabilizing Transformation) for five of the 1996 genes are plotted against dpi, with linear regression lines and 95% confidence intervals. Pearson correlation coefficients (r), coefficients of determination (r^2^) and corrected significances, FDR (q), are provided for each gene. **d** Using cellular deconvolution (as in Fig 3B and 3C), cell types whose abundance scores significantly positive correlated with dpi were identified. Linear regression lines and 95% confidence intervals, and Pearson correlation coefficients (r), coefficients of determination (r^2^) and corrected significances, FDR (q) are provided. (Full data sets are provided in S6 Table).

Enrichr and IPA analyses of the 2786 genes indicated a significant association with ciliated epithelial cells and protein synthesis, with 59 of the 2786 representing ribosomal protein genes (Fig 6A and S6 Table). Ciliated epithelial cells are a known target of SARS-CoV-2 infection [64,121], with omicron variants showing a higher propensity to infect these cells [76]. The increase in ciliated epithelial cell genes thus indicates growth/recovery and differentiation of these cells over the 2–66 days of the experiment. The increase in signatures associated with protein translation during this period is consistent with the development of adaptive immune responses and ongoing tissue repair processes.

### Genes whose expression negatively correlated with dpi indicated inflammation resolution

Using the complete gene sets, genes were identified whose expression showed significant negative correlation with dpi (Pearson correlations, q<0.05). Genes whose expression also showed significant negative correlation with days post inoculation of UV-inactivated virus were excluded from the analysis, thereby retaining only genes associated with active infection. The process identified 1996 genes (S6 Table).

IPA analyses of the 1996 genes identified reductions in cell movement/migration (Fig 6B), consistent with Figs 2A, 2B and 3A. A series of cytokine USRs were also down-regulated (S6 Table), with the top cytokine USRs (by negative z score) representing cytokines previously identified as being robustly associated with COVID-19 disease severity [11]. The latter was supported by identification of “Pathogen Induced Cytokine Storm Signaling Pathway” as a top IPA Canonical pathway annotation with a negative z score (Fig 6B). Also informative were decreasing neutrophil degranulation and fibrosis signatures (Fig 6B); both phenomena have been associated with long COVID [122,123].

Negative correlations between expression and dpi are plotted for five of the 1996 genes (Fig 6C). These genes were selected as their expression levels have been positively correlated with COVID-19 disease severity and/or they have been proposed as potential targets for prognosis or treatment; SerpinE1 [124], Cxcl10 [125], Nfkb1 [126], IL6 [127], and Hspa1b (a Hsp70 family member) [128,129].

### Cell types whose abundance scores increased with dpi indicated lung regeneration

Cell types whose abundance scores (determined by cellular deconvolution) showed a significant positive correlation (Pearsons q<0.05) with dpi were identified. The top scoring cell type was ciliated cells (Fig 6D); consistent with Fig 6A. The analysis also identified significant increases in AT1 cells, which likely reflects the AT2 to AT1 transition during alveolar epithelial cell regeneration seen after SARS-CoV-2 alveolar damage in mouse models [130]. Increasing abundance scores for reticular fibroblasts, are also consistent with successful lung repair [51,131], with no indication of significant fibrosis. Increasing numbers of innate lymphoid cells (ILC) likely also reflect the process of returning the tissue to homeostasis [132,133].

## Discussion

We describe herein the transcriptional profile of BA.1 omicron lung infections in K18-hACE2 mice, and the post-infection recovery up to 66 dpi. The expression of hACE2 via the keratin 18 promoter in the K18-hACE2 mice did not lead to overtly aberrant cell tropism in the lungs, with infection of bronchial and alveolar epithelia cells by omicron variants (Fig 1D and 1E) also described in settings where hACE2 is not expressed as a transgene [76,77]. Although there are some differences in methodologies, the overall consensus is that, when compared to infection with original strain isolates, BA.1 viral loads in lungs are lower and/or are more rapidly cleared in rodent models [74,75,134–137]. The BA.1 data presented herein (Fig 1A–1C) supports this contention when compared with previously published data on infection of K18-hACE2 mice with original strain isolates [55,64,73].

Herein we show that only a small number of DEGs were identified at 66 dpi with BA.1, arguing that the infection had been resolved and the tissue had largely returned to normal at this time. UV-inactivated virus was used as a control to exclude responses to viral inoculation and restrict the analyses to effects arising from replicating virus. Features often associated with post-acute or long-COVID were not identified, for instance, there were no indications of prolonged infection [138], persistent inflammation [139], impaired lung regeneration [140], fibrosis [123], prolonged T cell activation [141], Th17 bias [142], chronic neutrophil activation [122] or clotting abnormalities [8]. Only 8 mice were analyzed at > 30 dpi, which is arguably too low to identify a potential subset of individuals with post-acute or long-COVID [143,144]. Nevertheless, the overall picture that emerges for the BA.1 infection of K18-hACE2 is one of an attenuated, rapidly resolving, sequelae-free, infection. Importantly, lower viral loads and more rapid viral clearance are also associated in COVID-19 patients with less severe disease [35] and/or subclinical infections [46,47].

An inherent limitation with studies using K18-hACE2 mice is the level to which these animals recapitulate human infection and disease. For instance, fulminant lethal brain infections, usually seen 4–7 days after infection with original strain isolates, is not a feature of human disease [54,145,146]. However, such lethality is rare after BA.1 infections (S1b Fig in S1 File), thereby permitting the investigation of long-term responses presented herein. We have also shown that overlap between DEGs identified by RNA-Seq analysis of infected lung tissues from humans vs. K18-hACE2 mice is generally quite low; however, a high level of correlation emerged between inflammatory pathways [55]. Key clinical parameters used to evaluate disease severity in COVID-19 patients (e.g. oxygen saturation, respiration rate, chest imaging, full blood works) are also not readily available for mice in BSL3/PC3 settings. In addition, although lung samples are easily obtained from infected mice, they are not so readily available from COVID-19 patients. Finally, the plethora of different virus isolates, virus doses, rodent models (including age and gender), and analytical techniques, complicates detailed comparisons across studies [147–150]. For instance, although RNA-Seq data suggests increased pathogenicity of delta variants over ancestral (original) strain isolates in K18-hACE2 mice [148], numerous methodological differences complicate integration of such data into compelling comparisons with our study. Finally, it remains unclear to what extent inbred syngeneic mouse strains (with specific genetic backgrounds [63]) can faithfully recapitulate the activity of key genetic traits (e.g. HLA-DQA2) that underpin rapid SARS-CoV-2 clearance in humans [40].

The concept of “protective inflammation” was recently described for COVID-19 [56] and clearly applies to many other viral infections where subclinical or mild infections are also well documented [151]. Deleterious or pathological inflammatory disease processes tend to be the focus of research, with perhaps less attention paid to characterisation of inflammatory responses that are resolved and lead to subclinical or mild disease and sequelae-free outcomes [152,153]. Pathological versus protective responses are likely to have distinct gene expression profiles, patterns, magnitudes and sequence of events [57,58], with the current study describing the responses associated with resolution of lung infection and inflammation. Importantly, at 10 dpi a series of cell types associated with damage control, suppression of inflammation, homeostasis and repair showed increased abundance scores (determined using transcription data from lung tissues and cellular deconvolution). Specifically these cells were, ILC2 cells [86,87] (also seen at 5 dpi), inducible T regs [92,93], interstitial macrophages [94] and Ear2+ macrophages [95]. Restoration of ciliated cells, regeneration of alveolar epithelial cell with AT2 to AT1 transition [130], and increases in fibroblast activity without fibrosis [51], also appear to characterize lung recovery in this model. Clearly, prompt clearance of virus [46,50] (rather than viral persistence [138,154]) and development of protective adaptive immune responses [49] are also likely to be key to such outcomes. The latter has recently been shown to include CD8 T cells [96], which may be represented by a population of CD8b1^high^ cells which reached significance by 10 dpi in the BA.1/K18-hACE2 model.

Defining key aspects of “protective inflammation” remains a challenge for human populations, as individuals with subclinical or mild disease are generally difficult to identify and recruit promptly. A better understanding of what characterizes rapid, sequelae-free resolution of inflammation, would clearly facilitate development of interventions for COVID-19 associated ARDS, with animal models providing ready access to lung tissues to help unravel the complex mechanisms that underpin such processes.

## Supporting information

S1 File Supplementary figures 1 to 5. (PDF)

S1 Table Differential expression and pathway analysis for 2 dpi. (XLSX)

S2 Table Differential expression and pathway analysis for 5 dpi. (XLSX)

S3 Table Differential expression and pathway analysis for 10 dpi. (XLSX)

S4 Table Differential expression and pathway analysis for 30 dpi. (XLSX)

S5 Table Differential expression and pathway analysis for 66 dpi. (XLSX)

S6 Table Gene expression vs dpi. (XLSX)

## References

[1] Crits-Christoph A, Levy JI, Pekar JE, Goldstein SA, Singh R, Hensel Z, et al. Genetic tracing of market wildlife and viruses at the epicenter of the COVID-19 pandemic. Cell. 2024;187(19):5468–82.e11. doi: 10.1016/j.cell.2024.08.010 39303692 PMC11427129

[2] WHO COVID-19 dashboard 2023 [cited 2024 9 January]. Available from: https://data.who.int/dashboards/covid19/cases.

[3] WHO COVID-19 dashboard. https://data.who.int/dashboards/covid19/deaths?n=c. Accessed Feb 2024.

[4] Silva MJ, Ribeiro LR, Gouveia MI, Marcelino BD, Santos CS, Lima KV, et al. Hyperinflammatory Response in COVID-19: A Systematic Review. Viruses. 2023;15(2):553. doi: 10.3390/v15020553 36851766 PMC9962879

[5] Verma G, Dhawan M, Saied AA, Kaur G, Kumar R, Emran TB. Immunomodulatory approaches in managing lung inflammation in COVID-19: A double-edge sword. Immunity, Inflammation and Disease. 2023;11(9):e1020. doi: 10.1002/iid3.1020 37773723 PMC10521379

[6] Al-Nesf MAY, Abdesselem HB, Bensmail I, Ibrahim S, Saeed WAH, Mohammed SSI, et al. Prognostic tools and candidate drugs based on plasma proteomics of patients with severe COVID-19 complications. Nature Communications. 2022;13(1):946. doi: 10.1038/s41467-022-28639-4 35177642 PMC8854716

[7] Mueller MR, Ganesh R, Hurt RT, Beckman TJ. Post-COVID Conditions. Mayo Clinic Proceedings. 2023;98(7):1071–8. doi: 10.1016/j.mayocp.2023.04.007 37419575

[8] Altmann DM, Whettlock EM, Liu S, Arachchillage DJ, Boyton RJ. The immunology of long COVID. Nature Reviews Immunology. 2023;23(10):618–34. doi: 10.1038/s41577-023-00904-7 37433988

[9] Davis HE, McCorkell L, Vogel JM, Topol EJ. Long COVID: major findings, mechanisms and recommendations. Nat Rev Microbiol. 2023;21(3):133–46. doi: 10.1038/s41579-022-00846-2 . 36639608 PMC9839201

[10] Sick and tired: Casting a long shadow. Report April 2023. https://www.aph.gov.au/Parliamentary_Business/Committees/House/Health_Aged_Care_and_Sport/LongandrepeatedCOVID/Report. Accesses Feb 2024.

[11] Nasrollahi H, Talepoor AG, Saleh Z, Eshkevar Vakili M, Heydarinezhad P, Karami N, et al. Immune responses in mildly versus critically ill COVID-19 patients. Frontiers in Immunology. 2023;14:1077236. doi: 10.3389/fimmu.2023.1077236 36793739 PMC9923185

[12] Wang J, Jiang M, Chen X, Montaner LJ. Cytokine storm and leukocyte changes in mild versus severe SARS-CoV-2 infection: Review of 3939 COVID-19 patients in China and emerging pathogenesis and therapy concepts. Journal of Leukocyte Biology. 2020;108(1):17–41. doi: 10.1002/JLB.3COVR0520-272R 32534467 PMC7323250

[13] Qin R, He L, Yang Z, Jia N, Chen R, Xie J, et al. Identification of Parameters Representative of Immune Dysfunction in Patients with Severe and Fatal COVID-19 Infection: a Systematic Review and Meta-analysis. Clinical Reviews in Allergy & Immunology. 2023;64(1):33–65. doi: 10.1007/s12016-021-08908-8 35040086 PMC8763427

[14] Kalinina O, Golovkin A, Zaikova E, Aquino A, Bezrukikh V, Melnik O, et al. Cytokine Storm Signature in Patients with Moderate and Severe COVID-19. International Journal of Molecular Sciences. 2022;23(16):8879. doi: 10.3390/ijms23168879 36012146 PMC9408700

[15] Patel J, Bass D, Beishuizen A, Bocca Ruiz X, Boughanmi H, Cahn A, et al. A randomised trial of anti-GM-CSF otilimab in severe COVID-19 pneumonia (OSCAR). Eur Respir J. 2023;61(2):2101870. doi: 10.1183/13993003.01870-2021 . 36229048 PMC9558428

[16] Ghofrani Nezhad M, Jami G, Kooshkaki O, Chamani S, Naghizadeh A. The Role of Inflammatory Cytokines (Interleukin-1 and Interleukin-6) as a Potential Biomarker in the Different Stages of COVID-19 (Mild, Severe, and Critical). J Interferon Cytokine Res. 2023;43(4):147–63. doi: 10.1089/jir.2022.0185 . 37062817

[17] Mohd Zawawi Z, Kalyanasundram J, Mohd Zain R, Thayan R, Basri DF, Yap WB. Prospective Roles of Tumor Necrosis Factor-Alpha (TNF-&alpha;) in COVID-19: Prognosis, Therapeutic and Management. International Journal of Molecular Sciences. 2023;24(7):6142. 10.3390/ijms24076142. 10.3390/ijms24076142 PMC10094668 37047115

[18] Arunachalam PS, Wimmers F, Mok CKP, Perera R, Scott M, Hagan T, et al. Systems biological assessment of immunity to mild versus severe COVID-19 infection in humans. Science. 2020;369(6508):1210–20. doi: 10.1126/science.abc6261 . 32788292 PMC7665312

[19] Habel JR, Chua BY, Kedzierski L, Selva KJ, Damelang T, Haycroft ER, et al. Immune profiling of SARS-CoV-2 infection during pregnancy reveals NK cell and γδ T cell perturbations. JCI Insight. 2023;8(7):e167157. doi: 10.1172/jci.insight.167157 . 37036008 PMC10132165

[20] Carlini V, Noonan DM, Abdalalem E, Goletti D, Sansone C, Calabrone L, et al. The multifaceted nature of IL-10: regulation, role in immunological homeostasis and its relevance to cancer, COVID-19 and post-COVID conditions. Frontiers in Immunology. 2023;14:1161067. doi: 10.3389/fimmu.2023.1161067 37359549 PMC10287165

[21] Shih L-J, Yang C-C, Liao M-T, Lu K-C, Hu W-C, Lin C-P. An important call: Suggestion of using IL-10 as therapeutic agent for COVID-19 with ARDS and other complications. Virulence. 2023;14(1):2190650. doi: 10.1080/21505594.2023.2190650 36914565 PMC10026935

[22] Donlan AN, Sutherland TE, Marie C, Preissner S, Bradley BT, Carpenter RM, et al. IL-13 is a driver of COVID-19 severity. JCI Insight. 2021;6(15):e150107. doi: 10.1172/jci.insight.150107 . 34185704 PMC8410056

[23] Gibellini L, De Biasi S, Meschiari M, Gozzi L, Paolini A, Borella R, et al. Plasma Cytokine Atlas Reveals the Importance of TH2 Polarization and Interferons in Predicting COVID-19 Severity and Survival. Front Immunol. 2022;13:842150. doi: 10.3389/fimmu.2022.842150 . 35386702 PMC8979161

[24] Sasson J, Moreau GB, Petri WA. The role of interleukin 13 and the type 2 immune pathway in COVID-19: A review. Annals of Allergy, Asthma & Immunology. 2023;130(6):727–32. doi: 10.1016/j.anai.2023.03.009 36924937 PMC10014128

[25] Pourgholaminejad A, Pahlavanneshan S, Basiri M. COVID-19 immunopathology with emphasis on Th17 response and cell-based immunomodulation therapy: Potential targets and challenges. Scand J Immunol. 2022;95(2):e13131. doi: 10.1111/sji.13131 . 34936112

[26] Gupta P, Patel K, Garg G, Mathew BJ, Kale D, Singh J, et al. Imbalance of T Helper Cell Subset Specific Transcription Factors and Associated Cytokines in Patients with Severe COVID-19. Gene Expression. 2023;22(3):159–66. doi: 10.14218/GE.2023.00016

[27] Hasegawa T, Hato T, Okayama T, Ikeo K, Miyamoto Y, Iwanaga N, et al. Th1 cytokine endotype discriminates and predicts severe complications in COVID-19. European Cytokine Network. 2022;33(2):1–12. doi: 10.1684/ecn.2022.0477 36266985 PMC9595088

[28] Pavel AB, Glickman JW, Michels JR, Kim-Schulze S, Miller RL, Guttman-Yassky E. Th2/Th1 Cytokine Imbalance Is Associated With Higher COVID-19 Risk Mortality. Frontiers in Genetics. 2021;12:706902. doi: 10.3389/fgene.2021.706902 34335703 PMC8324177

[29] Gil-Etayo FJ, Garcinuño S, Utrero-Rico A, Cabrera-Marante O, Arroyo-Sanchez D, Mancebo E, et al. An Early Th1 Response Is a Key Factor for a Favorable COVID-19 Evolution. Biomedicines. 2022;10(2):296. doi: 10.3390/biomedicines10020296 35203509 PMC8869678

[30] Dhawan M, Rabaan AA, Alwarthan S, Alhajri M, Halwani MA, Alshengeti A, et al. Regulatory T Cells (Tregs) and COVID-19: Unveiling the Mechanisms, and Therapeutic Potentialities with a Special Focus on Long COVID. Vaccines. 2023;11(3):699. doi: 10.3390/vaccines11030699 36992283 PMC10059134

[31] Kosyreva A, Dzhalilova D, Lokhonina A, Vishnyakova P, Fatkhudinov T. The Role of Macrophages in the Pathogenesis of SARS-CoV-2-Associated Acute Respiratory Distress Syndrome. Frontiers in Immunology. 2021;12:682871. doi: 10.3389/fimmu.2021.682871 34040616 PMC8141811

[32] Meidaninikjeh S, Sabouni N, Marzouni HZ, Bengar S, Khalili A, Jafari R. Monocytes and macrophages in COVID-19: Friends and foes. Life Sciences. 2021;269:119010. doi: 10.1016/j.lfs.2020.119010 33454368 PMC7834345

[33] Dadras O, Afsahi AM, Pashaei Z, Mojdeganlou H, Karimi A, Habibi P, et al. The relationship between COVID-19 viral load and disease severity: A systematic review. Immunity, Inflammation and Disease. 2022;10(3):e580. doi: 10.1002/iid3.580 34904379 PMC8926507

[34] Waller H, Carmona-Vicente N, James A, Govender M, Hopkins FR, Larsson M, et al. Viral load at hospitalization is an independent predictor of severe COVID-19. Eur J Clin Invest. 2023;53(1):e13882. doi: 10.1111/eci.13882 . 36190270 PMC9874715

[35] Puhach O, Meyer B, Eckerle I. SARS-CoV-2 viral load and shedding kinetics. Nature Reviews Microbiology. 2023;21(3):147–61. doi: 10.1038/s41579-022-00822-w 36460930 PMC9716513

[36] Alene M, Yismaw L, Assemie MA, Ketema DB, Mengist B, Kassie B, et al. Magnitude of asymptomatic COVID-19 cases throughout the course of infection: A systematic review and meta-analysis. PLOS ONE. 2021;16(3):e0249090. doi: 10.1371/journal.pone.0249090 33755688 PMC7987199

[37] Oran DP, Topol EJ. The Proportion of SARS-CoV-2 Infections That Are Asymptomatic. Annals of Internal Medicine. 2021;174(5):655–62. doi: 10.7326/M20-6976 33481642 PMC7839426

[38] Polyak MJ, Abrahamyan L, Bego MG. Editorial: Immune determinants of COVID-19 protection and disease: A focus on asymptomatic COVID and long COVID. Front Immunol. 2023;14:1185693. doi: 10.3389/fimmu.2023.1185693 . 37056777 PMC10086613

[39] Sah P, Fitzpatrick MC, Zimmer CF, Abdollahi E, Juden-Kelly L, Moghadas SM, et al. Asymptomatic SARS-CoV-2 infection: A systematic review and meta-analysis. Proceedings of the National Academy of Sciences. 2021;118(34):e2109229118. doi: 10.1073/pnas.2109229118 34376550 PMC8403749

[40] Lindeboom RGH, Worlock KB, Dratva LM, Yoshida M, Scobie D, Wagstaffe HR, et al. Human SARS-CoV-2 challenge uncovers local and systemic response dynamics. Nature. 2024;631(8019):189–98. doi: 10.1038/s41586-024-07575-x . 38898278 PMC11222146

[41] Zuin M, Gentili V, Cervellati C, Rizzo R, Zuliani G. Viral Load Difference between Symptomatic and Asymptomatic COVID-19 Patients: Systematic Review and Meta-Analysis. Infectious Disease Reports. 2021;13(3):645–53. doi: 10.3390/idr13030061 34287354 PMC8293148

[42] Tsukagoshi H, Shinoda D, Saito M, Okayama K, Sada M, Kimura H, et al. Relationships between Viral Load and the Clinical Course of COVID-19. Viruses. 2021;13(2):304. doi: 10.3390/v13020304 33672005 PMC7919281

[43] Ra SH, Lim JS, Kim GU, Kim MJ, Jung J, Kim SH. Upper respiratory viral load in asymptomatic individuals and mildly symptomatic patients with SARS-CoV-2 infection. Thorax. 2021;76(1):61–3. doi: 10.1136/thoraxjnl-2020-215042 . 32963115

[44] Yin S-W, Zhou Z, Wang J-L, Deng Y-F, Jing H, Qiu Y. Viral loads, lymphocyte subsets and cytokines in asymptomatic, mildly and critical symptomatic patients with SARS-CoV-2 infection: a retrospective study. Virology Journal. 2021;18(1):126. doi: 10.1186/s12985-021-01597-x 34118952 PMC8197603

[45] Ooi EE, Low JG. Asymptomatic SARS-CoV-2 infection. The Lancet Infectious Diseases. 2020;20(9):996–8. doi: 10.1016/S1473-3099(20)30460-6 32539989 PMC7292578

[46] Bae S, Kim JY, Lim SY, Park H, Cha HH, Kwon J-S, et al. Dynamics of Viral Shedding and Symptoms in Patients with Asymptomatic or Mild COVID-19. Viruses. 2021;13(11):2133. doi: 10.3390/v13112133 34834940 PMC8625453

[47] Long Q-X, Tang X-J, Shi Q-L, Li Q, Deng H-J, Yuan J, et al. Clinical and immunological assessment of asymptomatic SARS-CoV-2 infections. Nature Medicine. 2020;26(8):1200–4. doi: 10.1038/s41591-020-0965-6 32555424

[48] De Biasi S, Tartaro DL, Gibellini L, Paolini A, Quong A, Petes C, et al. Endogenous control of inflammation characterizes pregnant women with asymptomatic or paucisymptomatic SARS-CoV-2 infection. Nature Communications. 2021;12(1):4677. doi: 10.1038/s41467-021-24940-w 34326336 PMC8322155

[49] Le Bert N, Clapham HE, Tan AT, Chia WN, Tham CYL, Lim JM, et al. Highly functional virus-specific cellular immune response in asymptomatic SARS-CoV-2 infection. Journal of Experimental Medicine. 2021;218(5):e20202617. doi: 10.1084/jem.20202617 33646265 PMC7927662

[50] Le Bert N, Samandari T. Silent battles: immune responses in asymptomatic SARS-CoV-2 infection. Cellular & Molecular Immunology. 2024;21(2):159–70. doi: 10.1038/s41423-024-01127-z 38221577 PMC10805869

[51] Soares-Schanoski A, Sauerwald N, Goforth CW, Periasamy S, Weir DL, Lizewski S, et al. Asymptomatic SARS-CoV-2 Infection Is Associated With Higher Levels of Serum IL-17C, Matrix Metalloproteinase 10 and Fibroblast Growth Factors Than Mild Symptomatic COVID-19. Front Immunol. 2022;13:821730. doi: 10.3389/fimmu.2022.821730 . 35479098 PMC9037090

[52] Sfikakis PP, Verrou KM, Ampatziadis-Michailidis G, Tsitsilonis O, Paraskevis D, Kastritis E, et al. Blood Transcriptomes of Anti-SARS-CoV-2 Antibody-Positive Healthy Individuals Who Experienced Asymptomatic Versus Clinical Infection. Front Immunol. 2021;12:746203. doi: 10.3389/fimmu.2021.746203 . 34675930 PMC8523987

[53] Ma Y, Deng J, Liu Q, Du M, Liu M, Liu J. Long-Term Consequences of Asymptomatic SARS-CoV-2 Infection: A Systematic Review and Meta-Analysis. Int J Environ Res Public Health. 2023;20(2):1613. doi: 10.3390/ijerph20021613 . 36674367 PMC9863678

[54] Stewart R, Yan K, Ellis SA, Bishop CR, Dumenil T, Tang B, et al. SARS-CoV-2 omicron BA.5 and XBB variants have increased neurotropic potential over BA.1 in K18-hACE2 mice and human brain organoids. Front Microbiol. 2023;14:1320856. doi: 10.3389/fmicb.2023.1320856 . 38075874 PMC10706942

[55] Bishop CR, Dumenil T, Rawle DJ, Le TT, Yan K, Tang B, et al. Mouse models of COVID-19 recapitulate inflammatory pathways rather than gene expression. PLoS Pathog. 2022;18(9):e1010867. doi: 10.1371/journal.ppat.1010867 . 36155667 PMC9536645

[56] Mangalmurti N, Hunter CA. Cytokine Storms: Understanding COVID-19. Immunity. 2020;53(1):19–25. doi: 10.1016/j.immuni.2020.06.017 . 32610079 PMC7321048

[57] Foy BH, Sundt TM, Carlson JCT, Aguirre AD, Higgins JM. Human acute inflammatory recovery is defined by co-regulatory dynamics of white blood cell and platelet populations. Nat Commun. 2022;13(1):4705. doi: 10.1038/s41467-022-32222-2 . 35995789 PMC9395541

[58] Doran AC, Yurdagul A, Tabas I. Efferocytosis in health and disease. Nature Reviews Immunology. 2020;20(4):254–67. doi: 10.1038/s41577-019-0240-6 31822793 PMC7667664

[59] Yan K, Dumenil T, Tang B, Le TT, Bishop CR, Suhrbier A, et al. Evolution of ACE2-independent SARS-CoV-2 infection and mouse adaption after passage in cells expressing human and mouse ACE2. Virus Evol. 2022;8(2):veac063. doi: 10.1093/ve/veac063 . 35919871 PMC9338707

[60] Morgan MS, Yan K, Le TT, Johnston RA, Amarilla AA, Muller DA, et al. Monoclonal Antibodies Specific for SARS-CoV-2 Spike Protein Suitable for Multiple Applications for Current Variants of Concern. Viruses. 2023;15(1):139. doi: 10.3390/v15010139 . 36680179 PMC9863740

[61] Rawle DJ, Le TT, Dumenil T, Yan K, Tang B, Nguyen W, et al. ACE2-lentiviral transduction enables mouse SARS-CoV-2 infection and mapping of receptor interactions. PLoS Pathog. 2021;17(7):e1009723. doi: 10.1371/journal.ppat.1009723 . 34214142 PMC8282004

[62] Yan K, Rawle DJ, Le TT, Suhrbier A. Simple rapid in vitro screening method for SARS-CoV-2 anti-virals that identifies potential cytomorbidity-associated false positives. Virol J. 2021;18(1):123. doi: 10.1186/s12985-021-01587-z . 34107996 PMC8188739

[63] Rawle DJ, Le TT, Dumenil T, Bishop C, Yan K, Nakayama E, et al. Widespread discrepancy in Nnt genotypes and genetic backgrounds complicates granzyme A and other knockout mouse studies. Elife. 2022;11:e70207. doi: 10.7554/eLife.70207 . 35119362 PMC8816380

[64] Dumenil T, Le TT, Rawle DJ, Yan K, Tang B, Nguyen W, et al. Warmer ambient air temperatures reduce nasal turbinate and brain infection, but increase lung inflammation in the K18-hACE2 mouse model of COVID-19. Sci Total Environ. 2022;859(Pt 1):160163. doi: 10.1016/j.scitotenv.2022.160163 . 36395835 PMC9659553

[65] TCID50_calculator_v2_17-01-20_MB ‐ Excel sheet to calculate TCID50 titers (Spearman & Kärber method). https://www.klinikum.uni-heidelberg.de/zentrum-fuer-infektiologie/molecular-virology/welcome/downloads. Accessed Feb 2024.

[66] Dagotto G, Mercado Noe B, Martinez David R, Hou Yixuan J, Nkolola Joseph P, Carnahan Robert H, et al. Comparison of Subgenomic and Total RNA in SARS-CoV-2-Challenged Rhesus Macaques. Journal of Virology. 2021;95(8): doi: 10.1128/JVI.02370-20 33472939 PMC8103707

[67] Schroder WA, Le TTT, Major L, Street S, Gardner J, Lambley E, et al. A Physiological Function of Inflammation-Associated SerpinB2 Is Regulation of Adaptive Immunity. The Journal of Immunology. 2010;184(5):2663–70. doi: 10.4049/jimmunol.0902187 20130210

[68] Bankhead P, Loughrey MB, Fernández JA, Dombrowski Y, McArt DG, Dunne PD, et al. QuPath: Open source software for digital pathology image analysis. Scientific Reports. 2017;7(1):16878. doi: 10.1038/s41598-017-17204-5 29203879 PMC5715110

[69] Bishop CR, Yan K, Nguyen W, Rawle DJ, Tang B, Larcher T, et al. Microplastics dysregulate innate immunity in the SARS-CoV-2 infected lung. Front Immunol. 2024;15:1382655. doi: 10.3389/fimmu.2024.1382655 . 38803494 PMC11128561

[70] Li S, Rouphael N, Duraisingham S, Romero-Steiner S, Presnell S, Davis C, et al. Molecular signatures of antibody responses derived from a systems biology study of five human vaccines. Nat Immunol. 2014;15(2):195–204. doi: 10.1038/ni.2789 . 24336226 PMC3946932

[71] Danaher P, Kim Y, Nelson B, Griswold M, Yang Z, Piazza E, et al. Advances in mixed cell deconvolution enable quantification of cell types in spatial transcriptomic data. Nat Commun. 2022;13(1):385. doi: 10.1038/s41467-022-28020-5 . 35046414 PMC8770643

[72] Livak KJ, Schmittgen TD. Analysis of Relative Gene Expression Data Using Real-Time Quantitative PCR and the 2−ΔΔCT Method. Methods. 2001;25(4):402–8. doi: 10.1006/meth.2001.1262 11846609

[73] Winkler ES, Bailey AL, Kafai NM, Nair S, McCune BT, Yu J, et al. SARS-CoV-2 infection of human ACE2-transgenic mice causes severe lung inflammation and impaired function. Nat Immunol. 2020;21(11):1327–35. doi: 10.1038/s41590-020-0778-2 . 32839612 PMC7578095

[74] Lee NY, Lee YW, Hong SM, On D, Yoon GM, An SH, et al. SARS-CoV-2 Omicron variant causes brain infection with lymphoid depletion in a mouse COVID-19 model. Lab Anim Res. 2023;39(1):8. doi: 10.1186/s42826-023-00157-4 . 37161442 PMC10169124

[75] Liu S, Selvaraj P, Sangare K, Luan B, Wang TT. Spike protein-independent attenuation of SARS-CoV-2 Omicron variant in laboratory mice. Cell Rep. 2022;40(11):111359. doi: 10.1016/j.celrep.2022.111359 . 36075211 PMC9420700

[76] Wu CT, Lidsky PV, Xiao Y, Cheng R, Lee IT, Nakayama T, et al. SARS-CoV-2 replication in airway epithelia requires motile cilia and microvillar reprogramming. Cell. 2023;186(1):112–30 e20. doi: 10.1016/j.cell.2022.11.030 . 36580912 PMC9715480

[77] Chiu MC, Li C, Liu X, Yu Y, Huang J, Wan Z, et al. A bipotential organoid model of respiratory epithelium recapitulates high infectivity of SARS-CoV-2 Omicron variant. Cell Discovery. 2022;8(1):57. doi: 10.1038/s41421-022-00422-1 35710786 PMC9203776

[78] Yinda CK, Port JR, Bushmaker T, Offei Owusu I, Purushotham JN, Avanzato VA, et al. K18-hACE2 mice develop respiratory disease resembling severe COVID-19. PLoS Pathog. 2021;17(1):e1009195. doi: 10.1371/journal.ppat.1009195 . 33465158 PMC7875348

[79] Amarilla AA, Sng JDJ, Parry R, Deerain JM, Potter JR, Setoh YX, et al. A versatile reverse genetics platform for SARS-CoV-2 and other positive-strand RNA viruses. Nat Commun. 2021;12(1):3431. doi: 10.1038/s41467-021-23779-5 . 34103499 PMC8187723

[80] Mills RJ, Humphrey SJ, Fortuna PRJ, Lor M, Foster SR, Quaife-Ryan GA, et al. BET inhibition blocks inflammation-induced cardiac dysfunction and SARS-CoV-2 infection. Cell. 2021;184(8):2167–82 e22. doi: 10.1016/j.cell.2021.03.026 . 33811809 PMC7962543

[81] Love MI, Huber W, Anders S. Moderated estimation of fold change and dispersion for RNA-seq data with DESeq2. Genome Biol. 2014;15(12):550. doi: 10.1186/s13059-014-0550-8 . 25516281 PMC4302049

[82] Falck-Jones S, Österberg B, Smed-Sörensen A. Respiratory and systemic monocytes, dendritic cells, and myeloid-derived suppressor cells in COVID-19: Implications for disease severity. Journal of Internal Medicine. 2023;293(2):130–43. doi: 10.1111/joim.13559 35996885 PMC9538918

[83] Lee MJ, Blish CA. Defining the role of natural killer cells in COVID-19. Nature Immunology. 2023;24(10):1628–38. doi: 10.1038/s41590-023-01560-8 37460639 PMC10538371

[84] von Borstel A, Nguyen THO, Rowntree LC, Ashhurst TM, Allen LF, Howson LJ, et al. Circulating effector γδ T cell populations are associated with acute coronavirus disease 19 in unvaccinated individuals. Immunology & Cell Biology. 2023;101(4):321–32. doi: 10.1111/imcb.12623 36698330

[85] Neill DR, Wong SH, Bellosi A, Flynn RJ, Daly M, Langford TKA, et al. Nuocytes represent a new innate effector leukocyte that mediates type-2 immunity. Nature. 2010;464(7293):1367–70. doi: 10.1038/nature08900 20200518 PMC2862165

[86] Fonseca W, Lukacs NW, Elesela S, Malinczak CA. Role of ILC2 in Viral-Induced Lung Pathogenesis. Front Immunol. 2021;12:675169. doi: 10.3389/fimmu.2021.675169 . 33953732 PMC8092393

[87] Pyung YJ, Park D-J, Kim CG, Yun C-H. Remodeling and Restraining Lung Tissue Damage Through the Regulation of Respiratory Immune Responses. Tissue Engineering and Regenerative Medicine. 2023;20(3):329–39. doi: 10.1007/s13770-022-00516-7 36763280 PMC9913030

[88] García M, Kokkinou E, Carrasco García A, Parrot T, Palma Medina LM, Maleki KT, et al. Innate lymphoid cell composition associates with COVID-19 disease severity. Clin Transl Immunology. 2020;9(12):e1224. doi: 10.1002/cti2.1224 . 33343897 PMC7734472

[89] Khaddaj-Mallat R, Aldib N, Bernard M, Paquette AS, Ferreira A, Lecordier S, et al. SARS-CoV-2 deregulates the vascular and immune functions of brain pericytes via Spike protein. Neurobiol Dis. 2021;161:105561. doi: 10.1016/j.nbd.2021.105561 . 34780863 PMC8590447

[90] Avolio E, Srivastava PK, Ji J, Carrabba M, Tsang CTW, Gu Y, et al. Murine studies and expressional analyses of human cardiac pericytes reveal novel trajectories of SARS-CoV-2 Spike protein-induced microvascular damage. Signal Transduct Target Ther. 2023;8(1):232. doi: 10.1038/s41392-023-01489-2 . 37268620 PMC10236384

[91] Yadav M, Louvet C, Davini D, Gardner JM, Martinez-Llordella M, Bailey-Bucktrout S, et al. Neuropilin-1 distinguishes natural and inducible regulatory T cells among regulatory T cell subsets in vivo. J Exp Med. 2012;209(10):1713–22, S1-19. doi: 10.1084/jem.20120822 . 22966003 PMC3457729

[92] Gladstone DE D’Alessio F, Howard C, Lyu M-A, Mock JR, Gibbs KW, et al. Randomized, double-blinded, placebo-controlled trial of allogeneic cord blood T-regulatory cells for treatment of COVID-19 ARDS. Blood Advances. 2023;7(13):3075–9. doi: 10.1182/bloodadvances.2022009619 36961352 PMC10043947

[93] Franco A, Song J, Chambers C, Sette A, Grifoni A. SARS-CoV-2 spike-specific regulatory T cells (Treg) expand and develop memory in vaccine recipients suggesting a role for immune regulation in preventing severe symptoms in COVID-19. Autoimmunity. 2023;56(1):2259133. doi: 10.1080/08916934.2023.2259133 37724524 PMC12167603

[94] Bain CC, MacDonald AS. The impact of the lung environment on macrophage development, activation and function: diversity in the face of adversity. Mucosal Immunol. 2022;15(2):223–34. doi: 10.1038/s41385-021-00480-w . 35017701 PMC8749355

[95] Chen J, Huang XR, Yang F, Yiu WH, Yu X, Tang SCW, et al. Single-cell RNA Sequencing Identified Novel Nr4a1(+) Ear2(+) Anti-Inflammatory Macrophage Phenotype under Myeloid-TLR4 Dependent Regulation in Anti-Glomerular Basement Membrane (GBM) Crescentic Glomerulonephritis (cGN). Adv Sci (Weinh). 2022;9(18):e2200668. doi: 10.1002/advs.202200668 . 35484716 PMC9218767

[96] Antoun E, Peng Y, Dong T. Vaccine-induced CD8+ T cells are key to protection from SARS-CoV-2. Nature Immunology. 2023;24(10):1594–6. doi: 10.1038/s41590-023-01621-y 37735590

[97] Yağcı S, Serin E, Acicbe Ö, Zeren Mİ, Odabaşı MS. The relationship between serum erythropoietin, hepcidin, and haptoglobin levels with disease severity and other biochemical values in patients with COVID-19. International Journal of Laboratory Hematology. 2021;43(S1):142–51. doi: 10.1111/ijlh.13479 33554466 PMC8014125

[98] Kandikattu HK, Venkateshaiah SU, Kumar S, Mishra A. IL-15 immunotherapy is a viable strategy for COVID-19. Cytokine & Growth Factor Reviews. 2020;54:24–31. doi: 10.1016/j.cytogfr.2020.06.008 32536564 PMC7537239

[99] Sadhu S, Dalal R, Dandotiya J, Binayke A, Singh V, Tripathy MR, et al. IL-9 aggravates SARS-CoV-2 infection and exacerbates associated airway inflammation. Nat Commun. 2023;14(1):4060. doi: 10.1038/s41467-023-39815-5 . 37429848 PMC10333319

[100] Kral M, van der Vorst EPC, Surnov A, Weber C, Döring Y. ILC2-mediated immune crosstalk in chronic (vascular) inflammation. Front Immunol. 2023;14:1326440. doi: 10.3389/fimmu.2023.1326440 . 38179045 PMC10765502

[101] Ghosn L, Chaimani A, Evrenoglou T, Davidson M, Grana C, Schmucker C, et al. Interleukin-6 blocking agents for treating COVID-19: a living systematic review. Cochrane Database Syst Rev. 2021;3(3):CD013881. doi: 10.1002/14651858.CD013881 . 33734435 PMC8406988

[102] Patterson BK, Seethamraju H, Dhody K, Corley MJ, Kazempour K, Lalezari J, et al. CCR5 inhibition in critical COVID-19 patients decreases inflammatory cytokines, increases CD8 T-cells, and decreases SARS-CoV2 RNA in plasma by day 14. International Journal of Infectious Diseases. 2021;103:25–32. doi: 10.1016/j.ijid.2020.10.101 33186704 PMC7654230

[103] Pius-Sadowska E, Niedźwiedź A, Kulig P, Baumert B, Sobuś A, Rogińska D, et al. CXCL8, CCL2, and CMV Seropositivity as New Prognostic Factors for a Severe COVID-19 Course. International Journal of Molecular Sciences. 2022;23(19):11338. doi: 10.3390/ijms231911338 36232655 PMC9570115

[104] Zizzo G, Cohen PL. Imperfect storm: is interleukin-33 the Achilles heel of COVID-19? Lancet Rheumatol. 2020;2(12):e779–e90. doi: 10.1016/S2665-9913(20)30340-4 . 33073244 PMC7546716

[105] Chen J, López-Moyado IF, Seo H, Lio C-WJ, Hempleman LJ, Sekiya T, et al. NR4A transcription factors limit CAR T cell function in solid tumours. Nature. 2019;567(7749):530–4. doi: 10.1038/s41586-019-0985-x 30814732 PMC6546093

[106] Odagiu L, May J, Boulet S, Baldwin TA, Labrecque N. Role of the Orphan Nuclear Receptor NR4A Family in T-Cell Biology. Front Endocrinol (Lausanne). 2020;11:624122. doi: 10.3389/fendo.2020.624122 . 33597928 PMC7883379

[107] Phelan DE, Shigemura M, Aldhafiri S, Mota C, Hall TJ, Sznajder JI, et al. Transcriptional Profiling of Monocytes Deficient in Nuclear Orphan Receptors NR4A2 and NR4A3 Reveals Distinct Signalling Roles Related to Antigen Presentation and Viral Response. Frontiers in Immunology. 2021;12:2447. doi: 10.3389/fimmu.2021.676644 34248958 PMC8267906

[108] Paudyal P, Shrestha S, Madanayake T, Shuster CB, Rohrschneider LR, Rowland A, et al. Grb7 and Filamin-a associate and are colocalized to cell membrane ruffles upon EGF stimulation. J Mol Recognit. 2013;26(11):532–41. doi: 10.1002/jmr.2297 . 24089360 PMC4096567

[109] Liu X, Wang X, Xu T, Liu X, Li L. Effects of Different Delivery Modes on the Expression of Vesicle Transport-Related Genes in Female Pelvic Floor Muscle Repair After Injury. Appl Biochem Biotechnol. 2023;196(2):667–78. doi: 10.1007/s12010-023-04510-0 . 37171760

[110] Li S, Wang Z, Chen M, Xiao Y, Min J, Hu M, et al. ArfGAP3 regulates vesicle transport and glucose uptake in myoblasts. Cell Signal. 2023;103:110551. doi: 10.1016/j.cellsig.2022.110551 . 36476390

[111] Zhang X, Pizzoni A, Hong K, Naim N, Qi C, Korkhov V, et al. CAP1 binds and activates adenylyl cyclase in mammalian cells. Proceedings of the National Academy of Sciences. 2021;118(24):e2024576118. doi: 10.1073/pnas.2024576118 34099549 PMC8214675

[112] Ding T, Zhou Y, Long R, Chen C, Zhao J, Cui P, et al. DUSP8 phosphatase: structure, functions, expression regulation and the role in human diseases. Cell & Bioscience. 2019;9(1):70. doi: 10.1186/s13578-019-0329-4 31467668 PMC6712826

[113] Schönfelder J, Seibold T, Morawe M, Sroka R, Schneider N, Cai J, et al. Endothelial Protein kinase D1 is a major regulator of post-traumatic hyperinflammation. Frontiers in Immunology. 2023;14:1093022. doi: 10.3389/fimmu.2023.1093022 36936923 PMC10017463

[114] Rivera J, Proia RL, Olivera A. The alliance of sphingosine-1-phosphate and its receptors in immunity. Nature Reviews Immunology. 2008;8(10):753–63. doi: 10.1038/nri2400 18787560 PMC2600775

[115] Tsui HS, Pham NVB, Amer BR, Bradley MC, Gosschalk JE, Gallagher-Jones M, et al. Human COQ10A and COQ10B are distinct lipid-binding START domain proteins required for coenzyme Q function. J Lipid Res. 2019;60(7):1293–310. doi: 10.1194/jlr.M093534 . 31048406 PMC6602128

[116] Mantle D, Heaton RA, Hargreaves IP. Coenzyme Q10 and Immune Function: An Overview. Antioxidants. 2021;10(5):759. doi: 10.3390/antiox10050759 34064686 PMC8150987

[117] Colomer-Molera M, Sastre D, Sole Codina L, Vallejo-Gracia A, Navarro Perez M, Capera-Aragones J, et al. Kv1.3-dependent immune system activation is regulated by KCNE4. Biophysical Journal. 2023;122(3):14a. doi: 10.1016/j.bpj.2022.11.302

[118] Li D, Jing J, Dong X, Zhang C, Wang J, Wan X. Activating transcription factor 3: A potential therapeutic target for inflammatory pulmonary diseases. Immunity, Inflammation and Disease. 2023;11(9):e1028. doi: 10.1002/iid3.1028 37773692 PMC10515505

[119] Palecanda A, Paulauskis J, Al-Mutairi E, Imrich A, Qin G, Suzuki H, et al. Role of the Scavenger Receptor MARCO in Alveolar Macrophage Binding of Unopsonized Environmental Particles. Journal of Experimental Medicine. 1999;189(9):1497–506. doi: 10.1084/jem.189.9.1497 10224290 PMC2193067

[120] Tabary M, Gheware A, Penaloza HF, Lee JS. The matricellular protein thrombospondin-1 in lung inflammation and injury. Am J Physiol Cell Physiol. 2022;323(3):C857–C65. doi: 10.1152/ajpcell.00182.2022 . 35912991 PMC9467471

[121] Schreiner T, Allnoch L, Beythien G, Marek K, Becker K, Schaudien D, et al. SARS-CoV-2 Infection Dysregulates Cilia and Basal Cell Homeostasis in the Respiratory Epithelium of Hamsters. Int J Mol Sci. 2022;23(9):5124. doi: 10.3390/ijms23095124 . 35563514 PMC9102945

[122] Woodruff MC, Bonham KS, Anam FA, Walker TA, Faliti CE, Ishii Y, et al. Chronic inflammation, neutrophil activity, and autoreactivity splits long COVID. Nature Communications. 2023;14(1):4201. doi: 10.1038/s41467-023-40012-7 37452024 PMC10349085

[123] Cui L, Fang Z, De Souza CM, Lerbs T, Guan Y, Li I, et al. Innate immune cell activation causes lung fibrosis in a humanized model of long COVID. Proceedings of the National Academy of Sciences. 2023;120(10):e2217199120. doi: 10.1073/pnas.2217199120 36848564 PMC10013740

[124] Kellici TF, Pilka ES, Bodkin MJ. Therapeutic Potential of Targeting Plasminogen Activator Inhibitor-1 in COVID-19. Trends Pharmacol Sci. 2021;42(6):431–3. doi: 10.1016/j.tips.2021.03.006 . 33867130 PMC7997307

[125] Lorè NI, De Lorenzo R, Rancoita PMV, Cugnata F, Agresti A, Benedetti F, et al. CXCL10 levels at hospital admission predict COVID-19 outcome: hierarchical assessment of 53 putative inflammatory biomarkers in an observational study. Molecular Medicine. 2021;27(1):129. doi: 10.1186/s10020-021-00390-4 34663207 PMC8521494

[126] Kircheis R, Haasbach E, Lueftenegger D, Heyken WT, Ocker M, Planz O. NF-κB Pathway as a Potential Target for Treatment of Critical Stage COVID-19 Patients. Frontiers in Immunology. 2020;11:598444. doi: 10.3389/fimmu.2020.598444 33362782 PMC7759159

[127] Nikkhoo B, Mohammadi M, Hasani S, Sigari N, Borhani A, Ramezani C, et al. Elevated interleukin (IL)-6 as a predictor of disease severity among Covid-19 patients: a prospective cohort study. BMC Infectious Diseases. 2023;23(1):311. doi: 10.1186/s12879-023-08294-w 37161412 PMC10169099

[128] Zangeneh Z, Khamisipour G. Elevated HSP70 and HSP90 as Predictive Markers of Immune Activation and Lung Injury in SARS-COV-2 Disease. Iranian Journal of Immunology. 2023;20(3):368–73. doi: 10.22034/iji.2023.97202.2488 37606151

[129] Prerna J, Swati G, Shailendra M, Kamini J, Manisha M, Rumaisha S, et al. Host inducible-HSP70A1A is an irresistible drug target to combat SARS-CoV2 infection and pathogenesis. bioRxiv. 2023:2023.05.05.539661. doi: 10.1101/2023.05.05.539661

[130] Dinnon KH, Leist SR, Okuda K, Dang H, Fritch EJ, Gully KL, et al. SARS-CoV-2 infection produces chronic pulmonary epithelial and immune cell dysfunction with fibrosis in mice. Science Translational Medicine. 2022;14(664):eabo5070. doi: 10.1126/scitranslmed.abo5070 35857635 PMC9273046

[131] Ghonim MA, Boyd DF, Flerlage T, Thomas PG. Pulmonary inflammation and fibroblast immunoregulation: from bench to bedside. J Clin Invest. 2023;133(17):e170499. doi: 10.1172/JCI170499 . 37655660 PMC10471178

[132] Monticelli LA, Sonnenberg GF, Abt MC, Alenghat T, Ziegler CG, Doering TA, et al. Innate lymphoid cells promote lung-tissue homeostasis after infection with influenza virus. Nat Immunol. 2011;12(11):1045–54. doi: 10.1031/ni.2131 . 21946417 PMC3320042

[133] Gomes AMC, Farias GB, Dias-Silva M, Laia J, Trombetta AC, Godinho-Santos A, et al. SARS-CoV2 pneumonia recovery is linked to expansion of innate lymphoid cells type 2 expressing CCR10. European Journal of Immunology. 2021;51(12):3194–201. doi: 10.1002/eji.202149311 34564853 PMC8646914

[134] Shuai H, Chan JF, Hu B, Chai Y, Yoon C, Liu H, et al. The viral fitness and intrinsic pathogenicity of dominant SARS-CoV-2 Omicron sublineages BA.1, BA.2, and BA.5. EBioMedicine. 2023;95:104753. doi: 10.1016/j.ebiom.2023.104753 . 37579626 PMC10448076

[135] Shuai H, Chan JF, Hu B, Chai Y, Yuen TT, Yin F, et al. Attenuated replication and pathogenicity of SARS-CoV-2 B.1.1.529 Omicron. Nature. 2022;603(7902):693–9. doi: 10.1038/s41586-022-04442-5 . 35062016

[136] Barut GT, Halwe NJ, Taddeo A, Kelly JN, Schön J, Ebert N, et al. The spike gene is a major determinant for the SARS-CoV-2 Omicron-BA.1 phenotype. Nature Communications. 2022;13(1):5929. doi: 10.1038/s41467-022-33632-y 36207334 PMC9543931

[137] Chen D-Y, Chin CV, Kenney D, Tavares AH, Khan N, Conway HL, et al. Spike and nsp6 are key determinants of SARS-CoV-2 Omicron BA.1 attenuation. Nature. 2023;615(7950):143–50. doi: 10.1038/s41586-023-05697-2 36630998

[138] Chen B, Julg B, Mohandas S, Bradfute SB, Force RMPT. Viral persistence, reactivation, and mechanisms of long COVID. eLife. 2023;12:e86015. doi: 10.7554/eLife.86015 37140960 PMC10159620

[139] Yin K, Peluso MJ, Luo X, Thomas R, Shin M-G, Neidleman J, et al. Long COVID manifests with T cell dysregulation, inflammation and an uncoordinated adaptive immune response to SARS-CoV-2. Nature Immunology. 2024;25:218–25. doi: 10.1038/s41590-023-01724-6 38212464 PMC10834368

[140] Zhao F, Ma Q, Yue Q, Chen H. SARS-CoV-2 Infection and Lung Regeneration. Clin Microbiol Rev. 2022;35(2):e0018821. doi: 10.1128/cmr.00188-21 . 35107300 PMC8809385

[141] Santopaolo M, Gregorova M, Hamilton F, Arnold D, Long A, Lacey A, et al. Prolonged T-cell activation and long COVID symptoms independently associate with severe COVID-19 at 3 months. eLife. 2023;12:e85009. doi: 10.7554/eLife.85009 37310006 PMC10319436

[142] De Biasi S, Meschiari M, Gibellini L, Bellinazzi C, Borella R, Fidanza L, et al. Marked T cell activation, senescence, exhaustion and skewing towards TH17 in patients with COVID-19 pneumonia. Nature Communications. 2020;11(1):3434. doi: 10.1038/s41467-020-17292-4 32632085 PMC7338513

[143] Di Gennaro F, Belati A, Tulone O, Diella L, Fiore Bavaro D, Bonica R, et al. Incidence of long COVID-19 in people with previous SARS-Cov2 infection: a systematic review and meta-analysis of 120,970 patients. Internal and Emergency Medicine. 2023;18(5):1573–81. doi: 10.1007/s11739-022-03164-w 36449260 PMC9709360

[144] Robertshaw M, Kershaw CD. Post COVID Interstitial Lung Abnormalities—Incidence and Management. Current Pulmonology Reports. 2023;12(2):64–9. doi: 10.1007/s13665-023-00307-y 37206298 PMC10098239

[145] Carossino M, Kenney D, O’Connell AK, Montanaro P, Tseng AE, Gertje HP, et al. Fatal Neurodissemination and SARS-CoV-2 Tropism in K18-hACE2 Mice Is Only Partially Dependent on hACE2 Expression. Viruses. 2022;14(3):535. doi: 10.3390/v14030535 35336942 PMC8955233

[146] Yang J-H, Yang M-S, Kim D-M, Kim B, Tark D, Kang S-M, et al. Delta (B1.617.2) variant of SARS-CoV-2 induces severe neurotropic patterns in K18-hACE2 mice. Scientific Reports. 2023;13(1):3303. doi: 10.1038/s41598-023-29909-x 36849513 PMC9970970

[147] Choi S, Lee J, Kim S, Lee YW, Kim GC, Hong SM, et al. A longitudinal molecular and cellular lung atlas of lethal SARS-CoV-2 infection in K18-hACE2 transgenic mice. EBioMedicine. 2024;99:104932. doi: 10.1016/j.ebiom.2023.104932 . 38118400 PMC10772566

[148] Lee KS, Wong TY, Russ BP, Horspool AM, Miller OA, Rader NA, et al. SARS-CoV-2 Delta variant induces enhanced pathology and inflammatory responses in K18-hACE2 mice. PLoS One. 2022;17(8):e0273430. doi: 10.1371/journal.pone.0273430 . 36037222 PMC9423646

[149] da Silva Santos Y, Gamon THM, de Azevedo MSP, Telezynski BL, de Souza EE, de Oliveira DBL, et al. Virulence Profiles of Wild-Type, P.1 and Delta SARS-CoV-2 Variants in K18-hACE2 Transgenic Mice. Viruses. 2023;15(4):999. doi: 10.3390/v15040999 . 37112979 PMC10146242

[150] Kim JA, Kim SH, Kim JJ, Noh H, Lee SB, Jeong H, et al. Immune Cells Are Differentially Affected by SARS-CoV-2 Viral Loads in K18-hACE2 Mice. Immune Netw. 2024;24(2):e7. doi: 10.4110/in.2024.24.e7 . 38725670 PMC11076298

[151] Rawle DJ, Hugo LE, Cox AL, Devine GJ, Suhrbier A. Generating prophylactic immunity against arboviruses in vertebrates and invertebrates. Nat Rev Immunol. 2024:(Online ahead of print). doi: 10.1038/s41577-024-01016-6 38570719

[152] Lee AC, Zhang AJ, Chan JF, Li C, Fan Z, Liu F, et al. Oral SARS-CoV-2 Inoculation Establishes Subclinical Respiratory Infection with Virus Shedding in Golden Syrian Hamsters. Cell Rep Med. 2020;1(7):100121. doi: 10.1016/j.xcrm.2020.100121 . 32984855 PMC7508015

[153] Everett HE, Lean FZX, Byrne AMP, van Diemen PM, Rhodes S, James J, et al. Intranasal Infection of Ferrets with SARS-CoV-2 as a Model for Asymptomatic Human Infection. Viruses. 2021;13(1):113. doi: 10.3390/v13010113 33467732 PMC7830262

[154] Proal AD, VanElzakker MB, Aleman S, Bach K, Boribong BP, Buggert M, et al. SARS-CoV-2 reservoir in post-acute sequelae of COVID-19 (PASC). Nature Immunology. 2023;24(10):1616–27. doi: 10.1038/s41590-023-01601-2 37667052

