# Supplementary material for: Tracking inflammation resolution signatures in lungs after SARS-CoV-2 omicron BA.1 infection of K18-hACE2 mice
Source: PLoS One. 2024 Nov 12;19(11):e0302344. doi: 10.1371/journal.pone.0302344 (PMC11556745; doi:10.1371/journal.pone.0302344)
Supplement: Attachment — Submitted filename: Response to reviewers comments.docx [file pone.0302344.s009.docx]

**Response to reviewers’ comments:** PONE-D-24-09241; Tracking inflammation resolution signatures in lungs after SARS-CoV-2 omicron BA.1 infection of K18-hACE2 mice.

Reviewers’ comments are reproduced in italics to facilitate re-review.

**Reviewer #2:** 

*Dear editor,*

*I reviewed the revised manuscript By Carolin et al (Review of revised PONE-D-24-09241) and read the authors responses.\*

*While I am satisfied with most of the corrections I would suggest that an additional issue be addressed:*

*The first revision included the following comment and the response follows:*

*Comment #4: These results are hard to translate to viral load/copy and do not account for genome/antigenome copies and it is recommended to apply real-time RT-PCR to covert the data to genome equivalents. Furthermore, the data in 1c show about 1 log higher copies compared to 1b – whereas the authors indicate that the loads at day 2 post infection are comparable (line 208).*

*Response: Fig. 1c has now been removed and references to previous data moved to the Discussion as requested (see 4 below). The key general contention, supported by a range of publications (now provided), is that BA.1 lung viral loads are lower and reduce faster than those seen after infection with original strain isolates. Our data is consistent with these previous reports for such comparisons in rodent models, with comparisons supported by viral titer and/or viral read data from RNA-Seq. These two measures provide independent viral load information. Side by side BA.1 vs. original strain qPCR evaluations of lung viral loads over time would provide suitable data, but would not change the aforementioned contention which is now very widely supported by a range of studies and a number of groups. Such evaluations also represents a considerable burden of work and sizable new animal experiments, which we feel are hard to justify in the face of such a body of existing evidence.*

*My response (and request) is as follows:*

*The idea was not to further demonstrate that viral titers decline rapidly in BA.1 infected mice and not to compare to the original strain but rather to use a reliable tool (qRT-PCR) for the data presented now in Fig. 1b . Instead of providing the RNA seq data of viral load analyses (in counts per million – a measure that cannot be translated to genomes/copies) – to analyze the same RNA (no need for more animals or BSL3 facility) by qRT-PCR to provide the data as viral copies/gr thus allowing a better comparison between Fig. 1 a and b.*

We have provided a side-by-side comparison between BA.1 and an original strain isolate using a RTqPCR based method to generate genome copy number data, as requested. This data is presented in a new Fig. 1c, with new sections for methods and results.
